# The role of spatial processing in verbal serial order working memory

**DOI:** 10.3758/s13415-024-01240-6

**Published:** 2025-01-15

**Authors:** Yingxue Tian, Simon Fischer-Baum

**Affiliations:** 1 Jefferson Moss Rehabilitation Research Institute, 50 Township Line Road, Elkins Park, PA 19027 USA; 2 Department of Psychological Sciences, Rice University, Houston, TX 77005 USA

**Keywords:** Serial order, Working memory, SPoARC effect, Multilevel modeling, Network neuroscience

## Abstract

In a sequence, at least two aspects of information—the identity of items and their serial order—are maintained and supported by distinct working memory (WM) capacities. Verbal serial order WM is modulated by spatial processing, reflected in the Spatial Position Association of Response Codes (SPoARC) effect—the left-beginning, right-end positional association between space and serial position of verbal WM memoranda. We investigated the individual differences in this modulation with both behavioral and neurobiological approaches. We administered a battery of seven behavioral tasks with 160 healthy adults and collected resting-state fMRI data from a subset of 25 participants. With a multilevel mixed-effects modeling approach, we found that the SPoARC effect’s magnitude predicts individual differences in verbal serial order WM capacity and is related to spatial item WM capacity. With a graph-theory-based analytic approach, this interaction between verbal serial order WM and spatial WM was corroborated in that the level of interaction between corresponding cortical regions (indexed by modularity) was predictive of the magnitude of the SPoARC effect. Additionally, the modularity of cortical regions associated with verbal serial order WM and spatial attention predicted the SPoARC effect’s magnitude, indicating the involvement of spatial attention in this modulation. Together, our findings highlight multiple sources of the interplay between verbal serial order WM and spatial processing.

**Supplementary information:**

The online version contains supplementary material available at 10.3758/s13415-024-01240-6.

## Introduction

To dial someone’s phone number, we need to keep track of all digits in the correct order; otherwise, even with all the correct digits, we might still dial the wrong number. When holding sequences in working memory (WM), we need to maintain at least two types of information—the item identities and their serial order. In recent years, there has been an increased interest in understanding how we are able to maintain serial order in WM. One prominent theory that has gained substantial attention is the “mental whiteboard hypothesis,” which states that this ability is supported by spatial representations that are repurposed to encode serial order as spatial information (Abrahamse et al., 2014, 2017). This theory has been corroborated by substantial and reproducible evidence supporting an association between serial order codes and spatial representation. However, we still do not know how this spatial processing truly supports our ability to maintain serial order in WM. In the current study, we investigate the role of spatial processing in verbal serial order WM, taking an individual differences approach to both behavioral and network neuroscientific data.

Separate capacities for serial order and item information in WM have been demonstrated in both the verbal (for review, see Majerus, 2019) and visuospatial domains (Claessen et al., 2016; Wansard et al., 2015), with further research indicating that serial order WM capacity is domain-specific (Tian et al., 2022, 2025).^1^ One behavioral phenomenon that has been repeatedly observed for sequences of verbal materials held in WM (Ginsburg et al., 2017; van Dijck et al., 2014) is the Spatial Position Association of Response Codes (SPoARC) effect, although strikingly it is not observed when sequences of spatial locations are held in WM (Ginsburg et al., 2017). When responding to information in a memorized list, there is a reaction time (RT) advantage of the left hand compared with the right hand for earlier items in the list; similarly, the left hand’s response is slower compared with the right hand when responding to later items in the memorized sequence. The mental whiteboard hypothesis (Abrahamse et al., 2014, 2017) accounts for this left-early, right-late mapping phenomenon, postulating that the memorized sequence is laid out from left to right along a mental "whiteboard”; thus, items at different serial positions are mapped onto different spatial locations and induce the differential RT patterns between hemifields of the responding hand. This spatialization process occurs for verbal sequences, and the claim is that verbal serial order WM is at least partially grounded in spatial processing.

The mental whiteboard hypothesis offers a compelling framework to explain the SPoARC effect but leaves several questions unanswered. First, although with prevalent observations of the SPoARC effect in verbal WM performance (Antoine et al., 2017; Bottini et al., 2016; Ginsburg et al., 2014, 2017; Guida et al., 2015, 2018a, 2020a, b; Hartmann et al., 2021; Rinaldi et al., 2015; van Dijck & Fias, 2011; van Dijck et al., 2022; Zhou et al., 2019, 2021), it remains unanswered whether the SPoARC effect has a functional role in verbal WM and facilitates the maintenance of serial order information. By treating verbal serial order WM as a capacity that varies amongst individuals, we can ask whether there is a relationship between the magnitude of the SPoARC effect and this capacity at the level of individual participants.

Second, it is unclear what spatial processing underlies the SPoARC effect. Again, using an individual differences approach, we can investigate which aspect(s) of spatial processing ability is associated with the magnitude of the SPoARC effect. Several candidate spatial processing abilities are considered. Two of these candidates are the exogenous and endogenous orienting of attention, which refer to the allocation of attention across space following salient stimuli or symbol-driven top-down modulation. It is possible that orienting attention in space is the underlying mechanism of the search process on the mental whiteboard. Indeed, a variety of results support a connection between these forms of attention and verbal WM. For example, verbal WM performance is enhanced when items are encoded at exogenously (Belopolsky et al., 2008) or endogenously cued locations (Huang & Pashler, 2007; Irwin & Gordon, 1998) compared with other locations and even when these locations are cued during the WM retention period (De Belder et al., 2015). The reverse is also true. During WM retention, when an item from the sequence is presented, attention is oriented faster to the location that corresponds to the serial position of the item compared with other locations (van Dijck et al., 2013, 2014). Another candidate for spatial processing ability is breadth of attention, which reflects the ability to spread the unitary focus of attention across space (Ball et al., 1988; Goodhew, 2020; Hüttermann et al., 2012, 2013; 2014; Kreitz et al., 2015; Pringle et al., 2001). Variability in breadth of attention has been found to be associated with WM performance in complex span (Bleckley et al., 2012) and n-back (Kreitz et al., 2015). In the context of the mental whiteboard hypothesis, the breadth of attention may reflect a limit of how much information can be spatially arrayed. As a result, a greater breadth of attention could manifest a broader mental whiteboard, allowing enhanced distinctiveness and better spatialization for verbal WM memoranda (Guida et al., 2017). The final set of candidates is spatial WM, rather than spatial attention. According to the mental whiteboard hypothesis, the SPoARC effect emerges from the proximity of the responding hand and the serial positions within the spatially recoded memorandum on the mental whiteboard. The spatial WM capacity to maintain spatial sequences (either the item or serial order information) could be the capacity that supports the recoded information on the mental whiteboard.

The current study is designed to address these open questions about the SPoARC effect and its relationship to WM capacity and spatial processing. Experiment 1 takes an individual differences approach with behavioral experiments. We used multilevel mixed-effects regression to characterize the relationship between the variability in the SPoARC effect, variability in item and serial order WM capacities for verbal and spatial information (Tian et al., 2022, 2025), and variability in spatial processing capacities like breadth of attention (Kreitz et al., 2015) and exogenous and endogenous orientation of attention (Posner, 1980). If, as is predicted by the mental whiteboard hypothesis, spatialization is a mechanism for verbal serial order WM, we predict that a larger SPoARC effect is associated with higher verbal serial order WM capacity, but not verbal item WM capacity. We also use this experiment to investigate which spatial processing underlies the mental whiteboard and supports the SPoARC effect, examining whether the variability in different kinds of spatial attention ability or spatial WM capacity correlates with variability in the SPoARC effect.

Experiment 2 takes on a similar question but instead focuses on how individual differences in the brain’s network structure relate to individual differences in the SPoARC effect. The logic of this experiment is as follows: The SPoARC effect is purportedly driven by interactions between verbal serial order WM and spatial processing. Previous research has identified brain regions that correspond to these distinct cognitive functions. The expectation is that the stronger the extent to which those brain regions are connected to each other—as measured through resting-state fMRI—the stronger the SPoARC effect. While prior work has evaluated the neural correlates of the SPoARC effect from task-based activations (Cristoforetti et al., 2022; Zhou et al., 2021), these studies have focused on behavior–brain relationship at a local level and fail to capture the interaction among brain regions.

Our individual differences approach to brain network structure focuses on the measure of network modularity, describing the degree to which a network is partitioned into modular structures while characterizing the interaction between modules (Newman, 2006). Like any network, the brain can be characterized as nodes (brain regions) and edges (the strength of connections between nodes), with graph theory providing tools and techniques for evaluating the structure of the network (Bassett & Sporns, 2017; Bullmore & Sporns, 2009; Medaglia, 2017; van den Heuvel & Hulshoff Pol, 2010). A network with high modularity can be described as having modules (clusters of nodes) that have strong edges within them, and weak or no edges to nodes in other modules. A network with low modularity has more (or stronger) edges with nodes in different modules and fewer (or weaker) edges with other nodes in the same module. A number of studies have taken an individual differences approach linking the modularity value of a brain network to performance on different cognitive abilities. Higher modularity values have been found to be associated with better performance in low-demand tasks (e.g., visual flanker task, color change detection) (Ramos-Nuñez et al., 2017; Yue et al., 2017), visual WM tasks (Stevens et al., 2012), spatial reasoning (Lebedev et al., 2018), as well as larger training effect in spelling training (Tao & Rapp, 2019), strategy-based gist reasoning training (Gallen et al., 2016), and WM training (Iordan et al., 2018), consistent with the idea that a high-modularity network is well-suited for specialized and automatized cognitive processes. In contrast, lower modularity values are associated with better performance in high-demand WM tasks (Kitzbichler et al., 2011), retrieval of episodic memory (Westphal et al., 2017), visual WM (Lebedev et al., 2018), and complex tasks (e.g., operation span, digit span, visual array, task shifting) (Ramos-Nuñez et al., 2017; Yue et al., 2017), consistent with the idea that a low-modularity network is more conducive to flexibly coordinating different regions for complex behaviors.

Modularity is particularly well-suited for the investigation of the SPoARC effect given the assumption that it is interactive in nature. We predict that a brain with a lower modularity value (i.e., with stronger connections between nodes in different modules) has stronger interactions between the distinct cognitive functions that give rise to the SPoARC effect and should yield a larger SPoARC effect. The question remains though, modularity over what brain network? Modularity measured over the whole brain may be too extensive and include too many nodes that are irrelevant to the task. Several studies have demonstrated that, compared with the whole brain, the modularity of more refined subnetworks has been reported to be associated with behavior more pronouncedly (Gallen et al., 2016; Iordan et al., 2018). Therefore, in Experiment 2, we included two scales of networks—the whole brain network and function-specific subnetworks—as candidates for the mesoscale neural correlates of the SPoARC effect. By investigating the relationship between modularity values of different networks and the magnitude of the SPoARC effect, we addressed the following questions in Experiment 2. (1) Does the modularity value of a network index the SPoARC effect in that a network of lower modularity implements a larger SPoARC effect? (2) If so, the network at which scale is better at predicting the variability of the SPoARC effect? Specifically, if spatial processing involved in the SPoARC effect is included in the subnetwork, we predict that the modularity value of the function-specific subnetwork is associated with the magnitude of the SPoARC effect to a greater extent than the whole brain network.

The results of these two experiments provide a neurocognitive account of the SPoARC effect, whose individual differences are modulated by different spatial processes. Ultimately, understanding the spatial underpinning of the SPoARC effect and the functional role it plays in verbal serial order WM could be the bridge to understanding the cognitive and neural architecture of verbal WM and its relationship to spatial processing.

## Experiment 1

Using multilevel mixed-effects model, we tested (1) whether the SPoARC effect facilitates serial order information in the verbal WM, but this facilitation does not apply to verbal item information nor spatial serial order information, and (2) which spatial processing—operationalized via spatial attention and spatial WM (focused on where locations are rather than their temporal context)—underlies the spatial recoding of verbal information and gives rise to the SPoARC effect.

### Method

#### Participants

A total of 182 participants were recruited from Rice University and Houston Community College. Participants were required to have normal or corrected-to-normal vision, normal hearing, no neurological abnormalities, no attentional disorders, no diagnosed reading and memory disorders, and able to make bimanual responses. All participants receive extra course credit or monetary compensation at the rate of $20 per hour for their participation. This study was approved by the Rice University Institutional Review Board.

Twenty-two participants were excluded from the final sample for the following reasons: participants did not meet the inclusion criteria (*n* = 3); participants had low accuracy in the catch trials (≤ 70%, which was the chance level of accuracy for 20 catch trials) in exogenous orienting of attention task (*n* = 8) and endogenous orienting of attention task (*n* = 6); participants had low task accuracy (≤ 56.6%, which was the chance level of accuracy for 168 trials) in endogenous orienting of attention task (*n* = 1); participants had low task accuracy (≤ 58.75%, which was the chance level of accuracy for 80 trials) in color item probe tasks (*n* = 13). A total of 22 participants were excluded: 14 met one criterion, 7 met two exclusion criteria, and 1 met three criteria.

The final sample size was 160 participants (87 females; age range = 18–23 years, mean = 19.4; 149 right-handed). All participants had English as their first language or as one of the simultaneous native languages. Six participants were exposed to languages with the reading/writing direction being right-to-left (e.g., Urdu, Arabic) or languages that are expressed in space (e.g., sign language).

#### Procedure

The consent form and demographic questionnaire (including age, gender, education level, and language background) were obtained from all participants upon arrival. Participants were seated individually in the testing room and tested for approximately 2 h. All tasks are computerized with E-Prime software (E-Prime 2.0, Psychology Software Tools, Inc., Pittsburgh, PA) and administered with Dell OptiPlex 9010 touchscreen PC in 1920 × 1080 resolution. Participants completed seven tasks in the following order: (1) breadth of attention; (2) exogenous orienting of spatial attention; (3) endogenous orienting of spatial attention; (4) item probe task with consonants; (5) verbal WM task; (6) spatial WM task; and (7) item probe task with colors. For participants who reported that they acquired right-to-left language(s) in the demographic questionnaire, a follow-up survey was conducted to document their proficiency after the completion of all tasks.

#### Tasks and materials

Seven tasks were administered in the current study. From five tasks, seven cognitive measures were calculated as level-2 (L2) variables, including three spatial attention measures (breadth of attention, exogenous orienting of spatial attention, endogenous orienting of spatial attention) and four WM measures (verbal item WM, verbal serial order WM, spatial item WM, spatial serial order WM). Two item probe tasks measured the WM performance for verbal and verbalizable visual materials and were used as the level-1 (L1) data for the multilevel model.

##### Breadth of attention (boa)

**Fig. 1:**
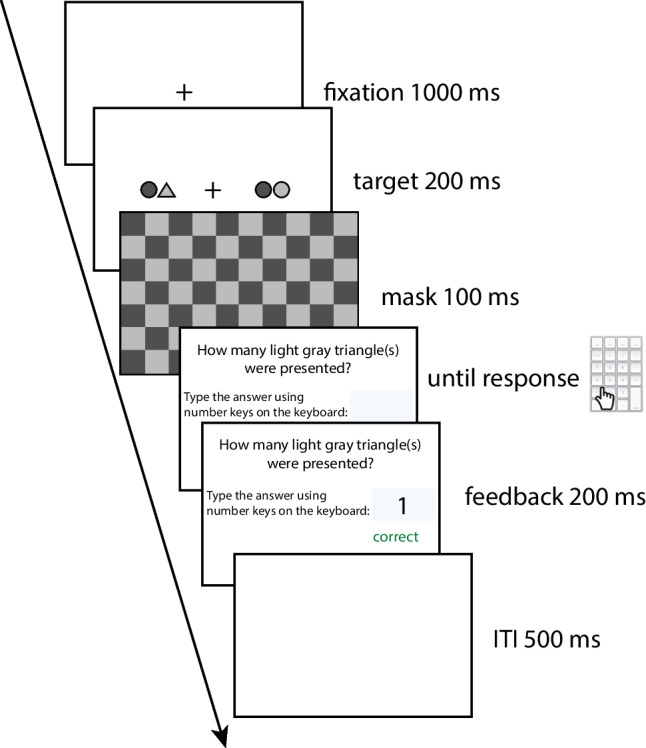
Schematic illustration of the breadth of attention task

The breadth of attention task was used to assess the spatial profile of the attention along the horizontal meridian, wherein participants can reliably monitor information of the visual field. In this task (adapted from Kreitz et al., 2015; see also Hüttermann et al., 2012, 2013, 2014), participants were instructed to fixate at the central fixation cross all the time and monitor stimuli on both sides of the screen simultaneously in the periphery. After familiarization, participants were given 132 experimental trials in this task. In each trial (Fig. 1), following a central fixation of 1000 ms, participants saw two clusters of objects (triangle/circle in light/dark gray) along the horizontal meridian at equidistance from the center for 200 ms and then a checkerboard mask for 100 ms. Afterward, participants reported the total number of light gray triangles with a number keypad. Given that the task required a conjunction search of features (both color and shape) about both clusters, the response required focused attention above perceptual processing (Schneider et al., 1982; Shiffrin & Schneider, 1977).

For each participant, trials were removed if their RTs were shorter than 100 ms, longer than 3000 ms, or outside the range of Q_1_ – 3 × IQR and Q_3_ + 3 × IQR (IQR = Q_3_ – Q_1,_ Q_1_ = first quantile, Q_3_ = third quantile of the distribution). With the trimmed dataset, accuracy at each distance from the center was calculated. The breadth of attention (*boa*) was calculated as the largest distance between two clusters beyond which participants did not correctly respond at higher than 75% accuracy (Clay et al., 2009; Hüttermann et al., 2012, 2014). It could range from 0 to 86.8 cm, with higher values reflecting a wider breadth of the attentional bottleneck.

The clusters of objects could appear at 33 plausible distances from the center of the display along the horizontal meridian, with the step size of 0.5 cm (0.7°). Each distance was tested four times.

##### Exogenous orienting of spatial attention (exo)

The exogenous orienting of spatial attention task assesses the efficiency of orienting attention in space following an exogenous cue. This task was adapted from the classic covert orienting visual attention task (COVAT) paradigm (Posner, 1980; Posner & Cohen, 1984) for reflexive orienting (Chica et al., 2013, 2014; Hayward & Ristic, 2013; McAuliffe & Pratt, 2005; Meyer et al., 2018).

**Fig. 2:**
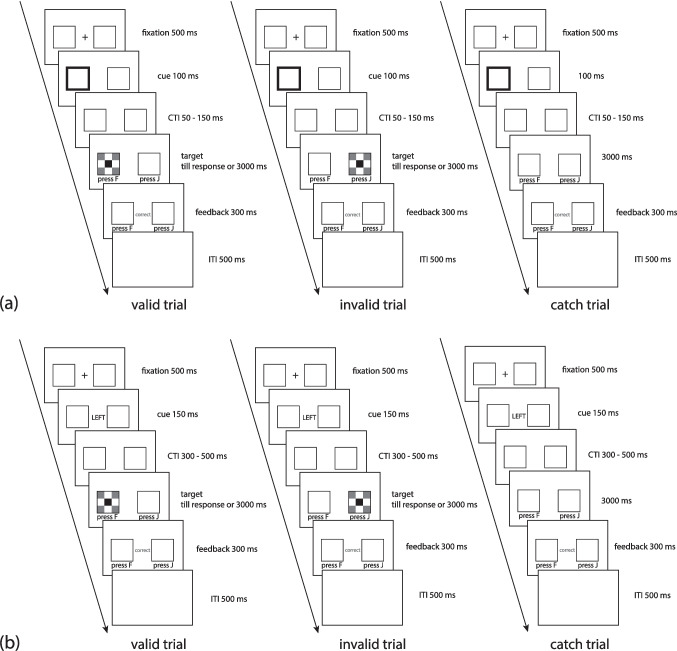
Schematic illustration for the (**a**) exogenous and (**b**) endogenous orienting of attention task

In this task, participants were informed of the predictivity of the cue (75% predictive) as well as the existence of the catch trials (i.e., when no target follows the cue). They were instructed to respond as quickly and accurately as possible but also refrain from responding in the catch trials. Participants were instructed to fixate in the center throughout the task so that there was no eye movement accompanying the covert orienting of attention. After familiarizing with the task and the predictivity of cues, there were 168 experimental trials in total, including 126 (75%) valid trials, 22 (13%) invalid trials, and 20 (12%) catch trials. Catch trials were included to discourage anticipatory response based simply on the lapse of time. In each trial, after a 500-ms fixation cross, one of the two peripheral placeholders flashed (changing the border width from 3 to 15 points) as the exogenous orienting cue for 100 ms. After a cue-target interval (CTI; 50–150 ms), a target appeared in one of the placeholders, and participants were instructed to press F or J on the keyboard as quickly as possible once they detected the target (Fig. 2a). The target appeared on the cued side in the valid trials and on the uncued contralateral side in the invalid trials. The target remained on the screen until a response was made or until 3000 ms had elapsed. No target appeared after the cue in catch trials. Feedback was provided after the response.

Participant-level outliers were excluded first for possible guessing behavior. If a participant responded to more than 30% of catch trials or had an accuracy of the task (across all trial types) lower than 56.6%, this participant was excluded from the subsequent analysis. The chance level of catch trials was determined as the accuracy of guessing performance on 20 catch trials (i.e., with a binomial distribution of *N* = 20 and *p* = 0.5 at α = 0.05). The chance level of task accuracy was calculated with a binomial distribution of *N* = 168 and *p* = 0.5 at α = 0.05. Eight participants had low accuracy in the catch trials and were excluded from subsequent analyses.

Correct noncatch trials in the task were used for analysis. For each participant, trials were removed if their RTs were extreme (shorter than 100 ms or longer than 1200 ms) or outside the range of Q_1_ – 3 × IQR and Q_3_ + 3 × IQR (IQR = Q_3_ – Q_1,_ Q_1_ = first quantile, Q_3_ = third quantile of the nonextreme correct noncatch trials’ RTs). Within the trimmed dataset, the cueing effect in exogenous orienting (*exo*) was calculated by subtracting the mean RT to detect a target in valid trials from the mean RT in invalid trials. A larger, positive value indicated a stronger efficiency in orienting with exogenous cues.

##### Endogenous orienting of spatial attention (endo)

A modified COVAT paradigm with endogenous orienting (Chica et al., 2014; Meyer et al., 2018; Posner, 1980; Posner & Cohen, 1984) was used to assess the efficiency of orienting attention in space following an endogenous cue. This task (Fig. 2b) was identical to the exogenous attention task, except that the duration of the cue was 150 ms, the cue was 75% predictive centrally presented symbolic cues (i.e., the word “left” or “right”), and the CTIs ranged from 300–500 ms (M = 388 ms) (Chica et al., 2014).

Participant-level outliers were excluded based on the same criteria as in the exogenous cueing task. Seven participants were excluded from further analysis owing to low accuracy in the catch trials (six participants) or low task accuracy (one participant). The cueing effect in endogenous orienting (*endo*) was calculated in the same manner as in the exogenous cueing task.

##### Verbal and spatial working memory tasks (viwm, vsowm, siwm, ssowm)

**Fig. 3:**
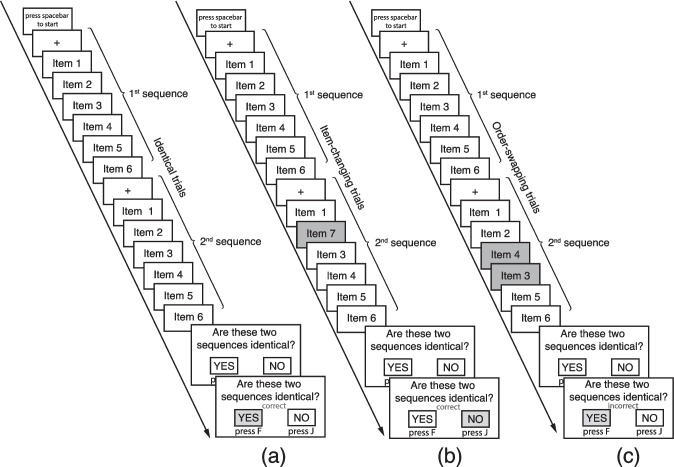
Schematic illustration for WM task: (**a**) identical trial, (**b**) item-changing trial, and (**c**) order-swapping trial

The sequence matching task (Tian et al., 2022a) was administered to measure item and serial order WM capacities in verbal and spatial domains, respectively. In each task, after familiarization, 120 trials were tested in a fixed order for all participants. In each trial, participants were presented with two consecutive six-item sequences and then instructed to answer, “Are these two sequences identical?” by pressing F for Yes and J for No on the keyboard. Three trial types are based on the match of two sequences. The second sequence was identical, one item changed, and two adjacent items swapped their serial positions compared with the first sequence (Fig. 3). Words and locations were used as items in verbal and spatial WM tasks, respectively. In the verbal WM task, each word was presented in the center of the screen for 800 ms. Words were selected from a set of 16 five-letter monosyllabic frequent nouns. The stimulus pool included four rhyming quadruplets, each sharing the same rime: /reɪn/ quadruplet (GRAIN, DRAIN, TRAIN, BRAIN), /ɪʧ/ quadruplet (PITCH, DITCH, HITCH, WITCH), /oʊst/ quadruplet (BOAST, COAST, ROAST, TOAST), and /aɪd/ quadruplet (BRIDE, GUIDE, PRIDE, SLIDE). In the spatial WM task, the default background of 16 static locations was presented throughout the entire presentation of a sequence, and each item in the sequence was indexed by one location turning solid black for 600 ms. The 16 locations were randomly distributed on the screen without clear verbal labels (e.g., exact position, collinearity, closure). Given the frequent use of verbal materials and the ease of recalling words compared with locations, we aimed to balance task difficulty by increasing the phonological similarity in the verbal stimulus pool while maintaining the same pool size across both domains.

Participant-level outliers were excluded for possible guessing behavior. If a participant had an accuracy of the task (across all trial types) lower than 57.5% and averaged RT in the task shorter than Q1 – 3 × IQR of the task RT distribution of all participants, this participant was excluded from the subsequent analysis. The chance level of task accuracy was calculated with a binomial distribution of *N* = 120 and *p* = 0.5 at α = 0.05. The conjunction criterion was adopted to remove potential guessing performance without excluding effortful performance. No participant met the conjunction criterion.

Nonidentical trials were used for subsequent analysis. For each participant, trials were removed if their RTs were extreme (shorter than 100 ms or longer than 3000 ms) or outside the range of Q_1_ – 3 × IQR and Q_3_ + 3 × IQR (IQR = Q_3_ – Q_1,_ Q_1_ = first quantile, Q_3_ = third quantile of nonextreme nonidentical trials’ RTs). Within the trimmed dataset for each task, the average accuracy of the item-changing and order-swapping trials were used as indices for item WM capacity (verbal item WM, *viwm,* and spatial item WM*, siwm*) and serial order WM capacity (verbal serial order WM, *vsowm,* and spatial serial order WM, *ssowm*), respectively.

##### Item probe tasks for the SPoARC effect

**Fig. 4:**
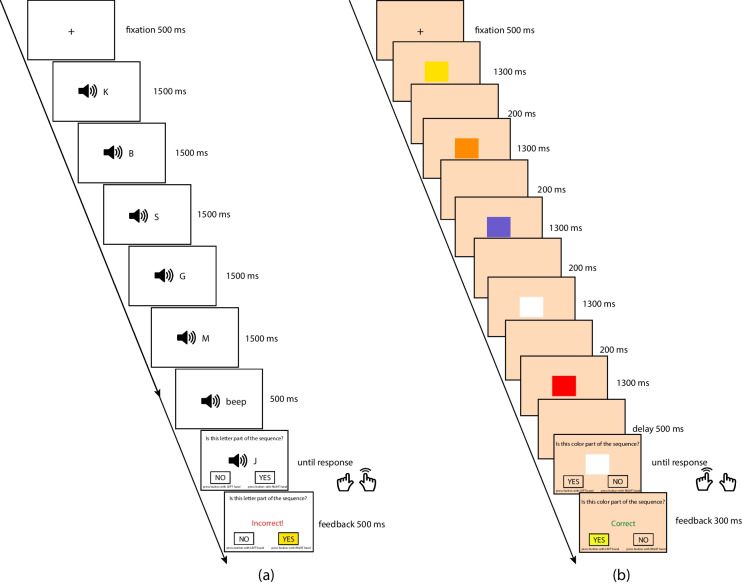
Item probe tasks for the elicitation of the SPoARC effect. (**a**) Trial with auditorily presented consonants with the left-NO-right-YES response mapping. (**b**) Trial with visually presented colors with the left-YES-right-NO response mapping

Item probe tasks with both auditorily presented consonants and visually presented colors (Guida et al., 2015, 2018b) were used to gauge a reliable and generalized SPoARC effect. In each trial, participants were presented with five items and then were asked to judge whether a probe was from the sequence by using two hands to press YES or NO corresponding to the response mapping requirement. Two response mapping was administered for each stimulus type, in the order of the left-NO, right-YES response mapping and then the left-YES, right-NO response mapping. These two mappings were administered in this fixed order. Each material type was tasked with 80 trials (40 trials for each response mapping). Figure 4 shows the scheme of the item probe task with both response mappings.

Eighteen consonants (all consonants except W, Y, and Z) were used for the consonant item probe task. Participants were instructed to put on headphones and adjust the output volume to their comfort level before the task started. Ten color patches over a light orange background were used for the color item probe task, including black, orange, blue, green, white, red, pink, yellow, purple, and gray. Ten colors can be categorized with clear verbal labels. Each item in the color item probe task was indexed as a solid-colored square in the center of the display for 1300 ms followed by a blank screen of 200 ms. The occurrence of each item at each serial position of the sequence and the frequency of each item being the probe were counterbalanced.

Participant-level outliers were excluded for possible guessing behavior. Accuracy for each stimulus type was calculated for each participant. If a participant had stimulus-wise accuracy lower than 58.75%, this participant was excluded from the subsequent analysis. The chance level of stimulus-wise accuracy was calculated with a binomial distribution of *N* = 80 and *p* = 0.5 at α = 0.05. No participant had low stimulus-wise accuracy in the consonant item probe task, and 13 participants had low stimulus-wise accuracy in the color item probe task.

For each participant, correct YES trials (i.e., trials with probed items from the memoranda) were trimmed for each material type separately. Trials were excluded if their RTs were extreme: shorter than 100 ms, longer than 3000 ms, or outside the range of Q_1_ – 3 × IQR and Q_3_ + 3 × IQR (IQR = Q_3_ – Q_1,_ Q_1_ = first quantile, Q_3_ = third quantile of nonextreme YES trials’ RTs). The trimmed datasets of both material types were combined as the level-1 dataset for the multilevel modeling analysis.

#### Multilevel mixed-effects model

In a repeated-measures study, the multilevel structure in the data is represented by repeated observations nested within individuals (Raudenbush & Bryk, 2002). In the current study, trials in two item probe tasks were nested within participants, who differed in their spatial attention and WM capacities. For a multilevel dataset, the multilevel models provide more accurate and robust estimates of parameters and variance partition than single-level ordinary least squares regression analyses (Bliese, 2002; Raudenbush & Bryk, 2002). Multilevel models also compute the best fit for a set of variables at different levels (i.e., trial’s characteristics, such as the interaction of hand and serial position, and participant’s characteristics, such as WM capacities and spatial attention capacities) simultaneously, which could avoid theoretically important effects being obscured.

In our multilevel model, 10,435 trials of consonant and color item probe tasks from 160 participants were included. Each participant had 44–79 trials (on average 66 trials). All trials’ RTs in two item probe tasks were modeled as the dependent variable, which was a function of *Hand* (the responding hand, 1 = right and –1 = left), *Position* (the serial position of the probe item*,* 1 to 5), the interaction *Hand* × *Position*, and two covariates *Task* (1 = consonant, 2 = color) and *Task* × *Hand* × *Position* of the trial. This constituted the first level (L1) of the model. At the second level (L2), we tested the main effects of twelve L2 variables, including seven cognitive variables (*boa*, *exo*, *endo*, *viwm*, *vsowm*, *siwm*, and *ssowm*) and five demographic covariates, which were *handedness* (1 = left-handed, 2 = right-handed), *altL*^2^ (*altL* = 1 when a participant acquired any language that is either read/written from right to left or expressed spatially), *education years*, *gender* (1 = male, 2 = female), and *school*. Most importantly, we included seven cross-level interactions (CLIs) in the multilevel model to assess moderating effects of seven cognitive variables on the effect of trial property *Hand* × *Position* on RT. A theory-driven four-way CLI *siwm* × *ssowm* × *Hand* × *Position* was also modeled,^3^ representing the concept that spatial item WM and spatial serial order WM interacted differently with the SPoARC effect. The by-participant random intercept was included in the model to capture the correlation among trials from the same participant. Three by-participant random slopes of *Hand* × *Position, Task, Task* × *Hand* × *Position* were also included. The significance of by-participant random slope of *Hand* × *Position* would validate the presence of individual differences of the SPoARC effect in item probe tasks. All L1 variables and L2 demographic control variables were uncentered and unstandardized; all L2 cognitive variables were grand-mean centered. All fixed and random effects were estimated simultaneously in the multilevel model (Raudenbush & Bryk, 2002).

The fit of a model was assessed by the deviance statistic (i.e., –2 × log-likelihood); a smaller deviance statistic suggested a better fit. AIC and BIC statistics complemented the deviance statistic of a model; smaller values implied a better fit to the data. The addition of predictors was assessed in a forward-fitting manner by comparing two nested multilevel models with and without these predictors. Building from the null model (i.e., the model with fixed and random intercepts of *participant* on the outcomes RT), five L1 predictors, twelve L2 predictors, three random slopes, and eight CLIs were added and assessed. Between two nested models, the difference in their deviance statistics (∆*D* = *D*_*1*_ – *D*_*2*_) has an approximate chi-squared distribution, where the degree of freedom is the difference between the degrees of freedom of two nested models (*df* = *p*_*2*_* – p*_*1*_). A significant chi-squared test endorses the more complex model. The final model with all aforementioned predictors was the best-fitting model (Table S1, Supplementary materials); we only reported results for the final model.

The fixed effects of CLIs were of critical interest because they directly assessed which, if any, spatial processing capacities’ differences could lead to different SPoARC effects in verbal WM performance. In addition, the CLI of *vsowm* × *Hand* × *Position* assessed whether verbal serial order WM differences moderate the SPoARC effect, elucidating if the SPoARC effect serves a functional role in keeping track of serial order information in verbal WM.

Significant CLIs were probed with simple slope tests (Aiken et al., 1991). The Johnson-Neyman regions of significance (Bauer & Curran, 2005; Curran et al., 2004; Johnson & Neyman, 1936; Preacher et al., 2006) were estimated for the L2 cognitive moderator, and pick-a-point approach (Rogosa, 1980) was used to probe the simple slopes at three levels (mean − SD, mean, and mean + SD) of the L2 moderator within its Johnson-Neyman regions of significance. At each level of the moderator, we used the differences of the simple slopes of *Position* between two hands to visualize the magnitude of the SPoARC effect. Note that this categorization is for illustrative purposes only; the statistical tests treated L2 cognitive variables as continuous variables.

### Results

The sample size for the following descriptive statistics and multilevel modeling analyses was 160.

#### Descriptive statistics and intercorrelations

Descriptive statistics for seven cognitive variables (before grand centering) are reported in Table 1. The skewness measures were less than 2, and the kurtosis measures were less than 4 for all seven cognitive variables, indicating that level-2 cognitive variables were approximately univariate normally distributed (Kline, 1998; Ryu, 2011).

**Table 1:** Descriptive statistics for level-2 cognitive variables

	Mean	*SD*	Min	Max	Skew	Kurtosis
*boa*	77.15	11.31	28.70	88.30	− 1.72	2.87
*exo*	33.54	34.49	− 64.01	132.05	0.50	0.57
*endo*	36.52	35.97	− 42.04	149.45	0.65	0.42
*viwm*	0.70	0.13	0.32	0.96	− 0.34	− 0.24
*vsowm*	0.65	0.18	0.21	0.97	− 0.41	− 0.69
*siwm*	0.81	0.16	0.29	1.00	− 1.10	0.59
*ssowm*	0.66	0.19	0.27	1.00	− 0.27	− 1.00

*N* = 160 for all variables. *boa* = breadth of attention; *exo* = efficiency of exogenous orienting; *endo* = efficiency of endogenous orienting; *viwm* = verbal item working memory capacity; *vsowm* = verbal serial order working memory capacity; *siwm* = spatial item working memory capacity; *ssowm* = spatial serial order working memory capacity.

Zero-order correlations among level-2 cognitive variables are reported in Table 2. *boa* was weakly associated with all other measures. Two orienting efficiencies in spatial attention (*exo* and *endo*) significantly correlated with each other moderately, suggesting that capacities to orient attention based on different nature of cues were related to some extent. Working memory measures correlated strongly within each content domain but only moderately across domains. All WM measures had negative and moderate correlations with exogenous orienting efficiency (*exo*) but were not correlated significantly with endogenous orienting efficiency (*endo*).

**Table 2:** Spearman correlation matrix of level-2 cognitive variables

	*boa*	*exo*	*endo*	*viwm*	*vsowm*	*siwm*
*exo*	.00					
*endo*	− .02	.20*				
*viwm*	.05	− .21*	.05			
*vsowm*	.03	− .22*	.03	.59*		
*siwm*	− .10	− .18*	.09	.32*	.30*	
*ssowm*	− .07	− .22*	.00	.31*	.40*	.66*

*N* = 160 for all variables. *Stands for significant correlations with *p* < .05. *boa* = breadth of attention; *exo* = efficiency of exogenous orienting; *endo* = efficiency of endogenous orienting; *viwm* = verbal item working memory capacity; *vsowm* = verbal serial order working memory capacity; *siwm* = spatial item working memory capacity; *ssowm* = spatial serial order working memory capacity.

#### Multilevel mixed-effects model

Table 3 reports the estimated parameters of the model and the fit of the model. The dataset included 10,435 level-1 data points (i.e., trials in the consonant and color item probe tasks), nested in 160 level-2 units (i.e., participants).

**Table 3:** Multilevel mixed-effects model for reaction time in item probe tasks

	Estimate (SE)
**Fixed effects**	
***L1***	
Intercept (γ_00_)	1210.44* (283.63)
* Hand* (γ_10_)	32.17* (6.6)
* Position* (γ_20_)	− 37.37 (1.92)
* Hand* × *Position* (γ_30_)	− 23.27* (4.02)
* Task* (γ_40_)	− 69.63* (13.94)
* Task* × *Hand* × *Position* (γ_50_)	11.27* (2.17)
***L2 cognitive variables***	
* boa* (γ_01_)	0.99 (1.34)
* exo* (γ_02_)	1.2* (0.5)
* endo* (γ_03_)	− 0.61 (0.45)
* viwm* (γ_05_)	58.34 (147.45)
* vsowm* (γ_06_)	183.36 (113.77)
* siwm* (γ_04_)	39.16 (133.9)
* ssowm* (γ_07_)	− 114.9 (113.24)
***L2 control variables***	
* Handedness* (γ_08_)	119.4* (58.7)
* altL* (γ_09_)	30.76 (78.23)
* School* (γ_010_)	7.68 (139.08)
* Education years* (γ_011_)	− 29.56 (15.14)
* Gender* (γ_012_)	66.99* (33.18)
***Cross-level interactions***	
* boa* × *Hand* × *Position* (γ_31_)	0.13 (0.1)
* exo* × *Hand* × *Position* (γ_32_)	− 0.02 (0.03)
* endo* × *Hand* × *Position* (γ_33_)	0.02 (0.03)
* viwm* × *Hand* × *Position* (γ_35_)	9.72 (10.36)
* vsowm* × *Hand* × *Position* (γ_36_)	− 19.37* (7.86)
* siwm* × *Hand* × *Position* (γ_34_)	− 24.25* (10.86)
* ssowm* × *Hand* × *Position* (γ_37_)	17.84* (7.95)
* siwm* × *ssowm* × *Hand* × *Position* (γ_347_)	− 100.39* (41.3)
**Random effects (variance components)**	
L1 variance (σ^2^)	76,098.40
Intercept (L2) variance (τ_00_)	100,786.00
* Hand* × *Position* (L2) variance (τ_33_)	1001.40
* Task* (L2) variance (τ_44_)	26,267.80
* Task* × *Hand* × *Position* (L2) variance (τ_55_)	357.20
Intercept–*Hand* × *Position* (L2) covariance (τ_03_)	− 5425.88
Intercept–*Task* (L2) covariance (τ_04_)	− 41,676.41
Intercept–*Task* × *Hand* × *Position* (L2) covariance (τ_05_)	3360.10
* Hand* × *Position*–*Task* (L2) covariance (τ_34_)	2256.99
* Hand* × *Position*–*Task* × *Hand* × *Position* (L2) covariance (τ_35_)	− 574.26
* Task*–*Task* × *Hand* × *Position* (L2) covariance (τ_45_)	− 1531.56
**Model summary (FIML)**	
ICC	0.29
Deviance statistic	147,888
AIC	147,962
BIC	148,231
Number of estimated parameters	37

L1 = Level 1; L2 = Level 2. L1 sample size = 10,435 and L2 sample size = 160. REML = restricted maximum likelihood estimation. All entries corresponding to the predicting variables are unstandardized estimations of the fixed effects γ_*ij*_ in the REML estimated model; values in parentheses are standard errors. *t*-statistics were computed as the ratio of each regression coefficient divided by its standard error. *p < .05 in a two-tailed *t*-test with the Satterthwaite approximation for their significance. FIML = full information maximum likelihood estimation; all fit indices (the deviance statistic, AIC, and BIC) were calculated from the FIML estimated model. *boa* = breadth of attention, *exo* = efficiency of exogenous orienting, *endo* = efficiency of endogenous orienting, *viwm* = verbal item WM capacity, *vsowm* = verbal serial order WM capacity, *siwm* = spatial item WM capacity, *ssowm* = spatial serial order WM capacity.

**Fig. 5:**
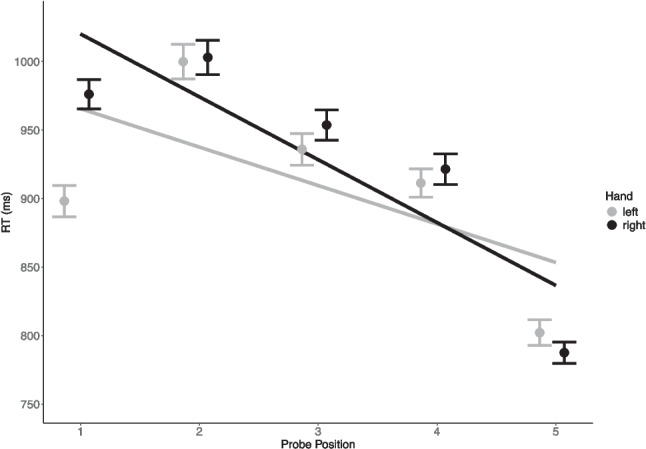
The SPoARC effect at the group level in item probe tasks from 160 participants. Each point is the averaged reaction time (RT) at the probed position in the sequence. Error bars represent standard error. Each line is the linear fit for reaction times as a function of the probed position in the sequence. Black = right hand, gray = left hand. A steeper line of the right hand than the left hand is indicative of the SPoARC effect

The multilevel model tested effects of level-1 trial characteristics, including *Hand*, *Position*, the interaction *Hand* × *Position* (i.e., the SPoARC effect), *Task*, and *Task* × *Hand* × *Position*. Compared with the null model, five level-1 predictors improved model fit significantly (${\upchi }^{2}$(5, *N* = 160) = 567.83, *p* < 0.001; Table S1, Supplementary materials). Specifically, the significant fixed effect of *Hand* × *Position* confirmed the presence of the SPoARC effect in item probe performance (γ_30_ = − 23.27, *t*(255.2) = 5.79, *p* < 0.05). This significant SPoARC effect replicates previous findings (Guida et al., 2015, 2018b) and reinforces the idea that serial order processing in verbal WM, at least in part, is operationalized through spatialization. We visualized the SPoARC effect at the group level (Fig. 5) by plotting averaged RT as a function of probed position for the left and right hand separately. A steeper slope for the right hand than the left hand was observed, indicating the presence of the SPoARC effect in item probe tasks.

The intraclass correlation (ICC) was calculated based on the null model to assess the need for multilevel modeling (Aguinis et al., 2013; Peugh, 2010). The ICC for the RTs of consonant and color item probe tasks was 0.293, indicating that 29.3% of the variance of RTs was attributable to between-participant differences and suggesting a multilevel analytic approach. Furthermore, the multilevel model assessed whether this variability in RT was accounted for by cognitive abilities or demographic differences with level-2 contextual effects. Twelve level-2 predictors (seven cognitive variables and five demographic variables) improved model fit significantly (${\upchi }^{2}$(12, *N* = 160) = 23.84, *p* = 0.02; Table S1, Supplementary materials).

Before moving on to the multilevel models testing for spatial processing’s moderation on the SPoARC effect, it is worthwhile to assess whether there was significant variability between individuals in the SPoARC effect in item probe tasks (reflected in the random slope of *Hand* × *Position* estimate). One useful property of the random-intercept-and-slopes multilevel model modeling technique is that it provides direct tests for between-participant variability, because the model allows each participant to have unique values for the *Hand* × *Position* estimates. Including random slopes for *Hand* × *Position*, *Task*, and *Task* × *Hand* × *Position* in the model led to significant model fit improvement, χ^2^(9, *N* = 160) = 626.11, *p* < 0.001 (Table S1, Supplementary materials). Specifically, between-participant variability in the magnitude of *Hand* × *Position* was significant, confirmed by additional chi-squared test for itself and nonparametric confidence intervals for the random slope of *Hand* × *Position* that excluded zero (see Supplementary materials)*.* This significant variance component confirmed reliable individual differences of the SPoARC effect in item probe tasks’ performance.

Most importantly, we used the multilevel model to estimate spatial attention and WM’s moderation of the between-participant variability of the SPoARC effect. Seven CLIs (the interaction of seven cognitive predictors and *Hand* × *Position*, respectively) and one theory-driven CLI *siwm* × *ssowm* × *Hand* × *Position* were assessed in the model simultaneously. The model fit improved significantly (${\upchi }^{2}$(8, *N* = 160) = 15.7,* p* = 0.046; Table S1, Supplementary materials), endorsing the addition of CLIs. Verbal serial order WM (*vsowm*), spatial item WM (*siwm*), and spatial serial order WM (*ssowm*) capacities significantly moderated the magnitude of the SPoARC effect: *vsowm* (γ_36_ = − 19.37, *t*(143.2) = 2.47, *p* = 0.02), *siwm* (γ_34_ = − 24.25, *t*(146.8) = 2.23, *p* = 0.03), and *ssowm* (γ_37_ = 17.84, *t*(143.3) = 2.25, *p* = 0.03). Furthermore, spatial item WM and spatial serial order WM moderated the SPoARC effect differently (*siwm* × *ssowm* × *Hand* × *Position*: γ_347_ = − 100.39, *t*(152.7) = 2.43, *p* = 0.02). The rest of CLIs were not significant.

To advance the interpretation of three significant three-way CLIs, we further probed them with simple slopes within Johnson-Neyman regions of significance of each level-2 cognitive variable, respectively. Both verbal and spatial serial order WM moderated the SPoARC effect without range limits, and spatial item WM significantly moderated the SPoARC effect only when it was not at the lower end (≥ 0.41 in the current sample of [0.29, 1], with 1% participants excluded).

**Fig. 6:**
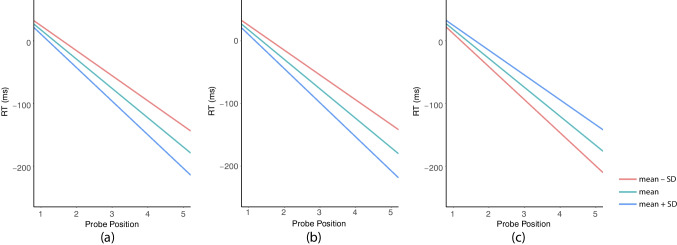
Simple slopes at three levels of moderator (a) verbal serial order WM, (b) spatial item WM, and (c) spatial serial order WM, respectively. Three levels are mean − SD (red), mean (green), and mean + SD (blue) of the moderator within its Johnson-Neyman region of significance; SD = standard deviation. The intercept and slope of each line are the difference between simple intercepts and simple slopes of *Position* subtracted from the right by the left hand, respectively. A negative slope indicates the presence of the SPoARC effect, and a steeper slope reflects a larger SPoARC effect

For verbal serial order WM (*vsowm*), three levels were selected, including 0.48, 0.65, and 0.82. As shown in Fig. 6a, as the verbal serial order WM capacity increased, the magnitude of the SPoARC effect (visualized as the difference in simple slopes of serial position between the right and left hand) increased. This pattern of CLI not only corroborates the extensive findings of the SPoARC effect being related to serial order processing in verbal WM but also indicates that the SPoARC effect serves a functional role in the maintenance of serial order information in verbal WM.

Opposite results were observed between two spatial WM capacities. For spatial item WM (*siwm*), three levels were selected within the Johnson-Neyman regions of significance (*siwm* ≥ 0.41), including 0.69, 0.82, and 0.97. As shown in Fig. 6b, participants with higher spatial item WM capacities showed larger SPoARC effects, suggesting that spatial item WM could be the spatial processing that supports the SPoARC effect. That is, participants who have high spans of spatial item WM could have greater cognitive capacity to support the spatialization of verbal memoranda on the mental whiteboard, and thus exhibit larger SPoARC effects than low-span participants.

For spatial serial order WM (*ssowm*), three levels were selected, including 0.48, 0.67, and 0.84. Figure 6c shows that as spatial serial order WM capacity increased, the magnitude of the SPoARC effect decreased. This pattern indicates that spatial serial order WM is not the behavioral underpinning of the SPoARC effect. Instead, the SPoARC effect might compete with spatial serial order WM for cognitive resources or interfere with its representation.

## Experiment 1 Discussion

The SPoARC effect is a robust behavioral phenomenon about serial order WM, and the current study investigates its behavioral underpinning and its function in the context of spatial attention and multi-faceted WM. We collected data for a seven-task behavioral battery from 160 participants. A multilevel mixed-effects model was used. RTs of two item probe tasks were level-1 data, and seven spatial attention and WM measures were level-2 variables of interest. We replicated previous findings of the SPoARC effect with single-level analytic approaches (Guida et al., 2015, 2018b) and confirmed reliable individual differences in the SPoARC effect (indexed by the significant random slope of *Hand* × *Position*). This is the first empirical evidence for reliable individual differences in the SPoARC effect.

The cross-level interactions in the multilevel model reveal how variability in the SPoARC effect relates to cognitive capacities, providing insights into the mechanism of how spatial processing moderates verbal WM. Notably, two positive relationships provide significant insights. First, participants exhibiting larger SPoARC effects (operationalized as the interaction of responding hand and serial position in verbal WM tasks) had higher verbal serial order WM capacities than participants with lower WM capacities, indicating that spatialization facilitates the maintenance of serial order information in verbal WM. Second, we operationalized spatial processing as three types of spatial attention and two types of spatial WM and found that larger SPoARC effect was associated with higher capacity to maintain item information in spatial WM. This finding suggests that spatial item WM is the behavioral underpinning of the SPoARC effect. Together, Experiment 1 provides a mechanistic understanding of the SPoARC effect.

## Experiment 2

Experiment 1 showed that spatial item WM, but not spatial attention, supports the SPoARC effect, and the SPoARC effect facilitates verbal serial order WM. In Experiment 2, we further assessed whether spatial attention is involved in the SPoARC effect with the network neuroscience approach. We examined (1) whether the modularity of a certain network is a valid mesoscale neural index for the SPoARC effect, and (2) the modularity at which scale of network is optimal to serve as the index. In Experiment 2, we examined the relationship between the magnitude of the SPoARC effect and the mesoscale network property modularity from resting-state neural activities at two different scales, including the whole brain network and two function-specific subnetworks. According to the mental whiteboard hypothesis, the SPoARC effect could arise from the interaction of verbal serial order WM and spatial attention. In addition, the results in Experiment 1 prompt us to examine the interaction of the network corresponding to verbal serial order WM, spatial item WM, and spatial serial order WM. Determining the optimal scale for the modularity can also elucidate what spatial processing is involved in the neural implementation supporting the SPoARC effect.

### Method

#### Participant

Twenty-five participants from Experiment 1 were recruited for Experiment 2 with additional neuroimaging data collected. All participants received extra course credit for their participation. This study was approved by the Rice University Institutional Review Board. Experiment 2 had a relatively small sample size, potentially limiting its statistical power. Nevertheless, this exploratory study offers a novel and promising perspective on understanding a behavioral phenomenon by examining the interaction between neural correlates of the cognitive processes involved. As an initial exploratory investigation, the findings would be further strengthened with a larger sample.

#### Procedure

All participants were tested in two separate sessions. The first session was a neuroimaging session, with both structural and functional MRI data obtained. This session lasted approximately 40 min. The second session was the behavioral session, which was administered within a week after the first session. The same battery of seven behavioral tasks as in Experiment 1 was administered in the second session, which took approximately 2 h. Only data from two item probe tasks were analyzed in Experiment 2.

#### Resting-state fMRI

##### Imaging data acquisition

Resting-state fMRI scans were conducted at the Core for Advanced Magnetic Resonance Imaging at Baylor College of Medicine. Images were obtained on a Siemens 3-Tesla Prisma Fit Scanner with a 64-channel transmit/receive head coil. Participants were instructed to stay still during the scanning, and foam pads were used to keep participants’ heads stable. A high-resolution T1-weighted structural image was acquired with magnetization prepared rapid gradient echo (MPRAGE) sequence in the sagittal direction (repetition time (TR) = 2400 ms, echo time (TE) = 2.22 ms, field of view (FOV) = 208 mm, voxel size = 2 × 2 × 2 mm^3^). Afterward, three seven-minute resting-state functional runs were obtained by using a gradient-echo echo-planar imaging (EPI) sequence as follows: TR = 1000 ms, TE = 35.4 ms, FOV = 208 mm, voxel size = 2 × 2 × 2 mm^3^, and multiband acceleration factor = 6. The anterior-to-posterior phase-encoding direction was used for functional run 1 and run 3, and the posterior-to-anterior phase-encoding direction was used for functional run 2.

##### Preprocessing

Preprocessing was conducted using the AFNI afni_proc.py (AFNI version: 20.0.18; Cox, 1996). Each functional run was preprocessed separately. For each run, the functional data was an EPI time series with 420 time points, with a 72-axial-slice volume at each time point; the signals being processed were the intensities of the BOLD (blood-oxygen-level-dependent) signal in each voxel at each slice.

Functional data was first preprocessed temporally, including de-spiking to reduce large fluctuations and slice timing correction. We then corrected for geometric distortion. Functional data were then preprocessed spatially, whereby head motions were corrected, and functional scans were aligned to each individual’s structural scan and warped to the Talairach-Tournoux standard space. Last, functional data was smoothed with a 3-mm FWHM Gaussian kernel, and a whole brain mask was created and applied to the functional data.

Following the preprocessing, the outlier censoring was conducted. A time point was removed when the Euclidean norm of head motion from the previous time point exceeded 0.2 or when more than 5% of whole brain voxels of a volume were outlier voxels. After censoring, a general linear model was applied to each voxel’s functional time series. 54 regressors were used in the model to regress out nuisance signals, including 12 Legendre polynomial regressors for baseline drifts (zero-, first-, second-, and third-degree polynomials for each run), 18 head motion regressors (for each run, rotations and translations in three axes) and 18 corresponding motion derivative regressors, and six tissue-based regressors (the first three principal components of the ventricles and white matter for all three runs). Additionally, an orthogonal voxel-wise regressor was used to remove signals from locally averaged white matter. After the regression, the residual time series were used for the subsequent network analyses.

##### Network analysis

The SPoARC effect arises from the interaction of verbal serial order WM and other spatial processing; therefore, we predict that the modularity of a network that characterizes this interaction between cortical regions could be a neural correlate for the SPoARC effect. However, the interactivity within the whole brain network might be too extensive to account for the variability of the SPoARC effect. Hence, we investigated networks at two scales, including the whole brain network and two function-specific subnetworks.

The SPoARC effect relates to verbal serial order WM performance and is postulated to be supported by spatial processing. Two function-specific subnetworks were of theoretical interest. According to the mental whiteboard hypothesis (Abrahamse et al., 2014, 2017), the SPoARC effect is grounded in spatial attention, and thus the first function-specific subnetwork was defined as the network of verbal serial order WM + spatial attention. According to the results in Experiment 1,^4^ the magnitude of the SPoARC effect is related to verbal serial order WM, spatial item WM, and spatial serial order WM. Hence, the second function-specific subnetwork was defined as the network of verbal serial order WM + spatial item WM + spatial serial order WM.

**Fig. 7:**
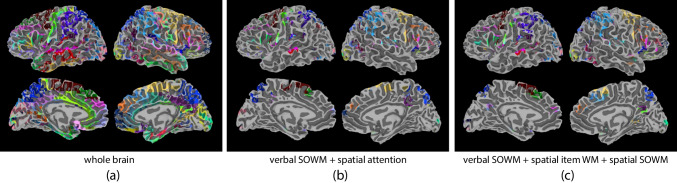
**(a) **Whole brain network, **(b)** function-specific subnetwork for verbal serial order WM and spatial attention, and **(c)** function-specific subnetwork for verbal serial order WM, spatial item WM, and spatial serial order WM. SOWM = serial order working memory. All networks are in the Brodmann atlas, with different colors representing distinct BA nodes

To construct the whole brain network, with the Talairach Daemon atlas in AFNI (AFNI version: AFNI_20.0.18; Cox, 1996), the whole brain was parcellated into 84 Brodmann areas (BAs, 42 BAs in each hemisphere).^5^ Each BA was used as a node for the network. For each node, a mean time series was calculated by averaging the preprocessed time series across all voxels within the given node. The edges between each pair of nodes in the network were defined as the functional connectivity, which is the Pearson correlation of the time series for two nodes. An adjacency matrix was calculated for each run separately. For each participant, the network was characterized by averaging across three adjacency matrices.^6^ An 84 × 84 adjacency matrix with 3486 edges was used to describe the network at the whole brain scale for each participant (Fig. 7a).

For function-specific subnetworks, we reviewed the literature for neural regions involved in four constituent cognitive functions, including verbal serial order WM, spatial item WM, spatial serial order WM, and spatial attention (both exogenous orienting and endogenous orienting). For each constituent function, peak coordinates of activation clusters reported in the task-based fMRI studies were used, and 8-mm radius spheres were created with the center being each peak coordinate. The mask for each constituent function was generated as the union mask of all spheres and then aligned to the Talairach Daemon atlas and parcellated into BAs. Masks of two function-specific subnetworks were created as the union mask of involved constituent cognitive functions’ masks (Figs. 7b and c). Previous studies have identified overlap between regions involved in spatial attention and spatial WM (Awh & Jonides, 2001; Labar et al., 1999). We observed this overlap in our function-specific masks, covering 41.4% of the verbal serial order WM + spatial attention network, and 11.1% of the verbal serial order WM + spatial item WM + spatial serial order WM network. Covered brain regions within each BA^7^ serve as nodes for the network. For each node, the mean time series was calculated by averaging across covered voxels in the node. Pairwise functional connectivity was calculated as edges for the network. An adjacency matrix was calculated for each run separately, and the mean adjacency matrix averaged across three runs was used for the participant’s network. For the function-specific subnetworks, the verbal serial order WM + spatial attention network was described by a 66 × 66 adjacency matrix with 2145 edges; the verbal serial order WM + spatial item WM + spatial serial order WM network was described by a 72 × 72 adjacency matrix with 2556 edges.

Cortical regions involved in each constituent cognitive function were identified from task-based fMRI studies as follows (Tables S3 to S6, Supplementary Materials). Our analysis focused on the interactivity between regions rather than specific areas. Therefore, to ensure comprehensive coverage, we adopted an inclusive approach in literature selection, aiming to minimize the risk of overlooking relevant regions. We included a wide range of tasks that engage the same cognitive process, while maintaining specificity in the cognitive process involved. For example, regions involved in serial order WM were identified using tasks that emphasize serial order processing relative to item processing or baseline conditions but not through tasks like immediate serial recall that involve both item and order processing (unless order information was explicitly decoded). Additionally, we cross-referenced our selections with studies included in Neurosynth (Yarkoni et al., 2011). For verbal serial order WM, search terms included “working memory”; “working”; “verbal working”; and “serial.” For spatial item and serial order WM, search terms included “working memory”; “working”; “visuospatial”; and “visuo-spatial.” For spatial attention, search terms included “spatial attention”; “endogenous”; “orienting”; “visuospatial”; and “visuo-spatial.” From the studies identified in Neurosynth, we retained studies that aligned with our target functions.

- **Verbal serial order WM**
  We reviewed task-based fMRI studies for the neural correlates of verbal serial order WM and created functional spherical ROIs based on reported peak activation coordinates (Table S3, Supplementary Materials). Cortical regions involved in verbal serial order WM were identified by stronger univariate activations in the serial order condition compared with the control or item condition (Attout et al., 2019; Attout et al., 2014a; Henson et al., 2000; Majerus et al., 2009; Majerus et al., 2008a, 2006c; Marshuetz et al., 2000, 2006; Öztekin et al., 2009) or by multivariate decoding of serial order representation (Kalm & Norris, 2014). This mask was aligned to the Brodmann atlas, and 52 nodes were covered.
  In the order probe task, participants saw or heard a sequence of verbal items and were probed with a question about the relative order between two items in the memoranda. When the verbal items were letters, compared with the neural activities during the item probe task, the left inferior parietal cortex, left IPS, right DLPFC, and right SFG were reported to have increased activation in order probe tasks (Marshuetz et al., 2006). In addition, stronger activations were found in the bilateral posterior parietal cortex (PPC), left IFG, and left supplementary motor area (SMA) (Marshuetz et al., 2000; Öztekin et al., 2009). Numerical processing-related regions (bilateral IPS) were also found in the contrast of serial order versus item WM conditions (Attout et al., 2014a; Marshuetz et al., 2000). When the verbal items were nonwords, the inferior frontal gyrus was found to be associated with verbal serial order WM in addition to orthographic (left mid-fusiform gyrus) and phonological processing (STG) regions (Majerus et al., 2009). When using words, similar results regarding bilateral SFG, MFG, and IPS were reported (Attout et al., 2019; Majerus et al., 2008a; Majerus, Poncelet, Van der Linden, et al., 2006c).
  In a similar paradigm, participants were asked to judge whether a sequence of letters with an order swap between items was identical to the memoranda. The left lateral premotor cortex and bilateral superior parietal gyrus were reported to be more active when detecting the order swaps than in the item probe condition (Henson et al., 2000).
  Based on opposing theoretical models of serial order, Kalm and Norris (2014) used representational similarity analysis to decode the neural correlates of serial order WM during immediate serial recall of nonword sequences. The superior planum temporale, inferior parietal lobule, SMG, and left ventral premotor cortex were above chance when decoding serial order information as positional tags.

- **Spatial item WM**
  We reviewed task-based fMRI studies for the neural correlates of spatial item WM and created functional spherical ROIs based on reported peak activation coordinates (Table S4, Supplementary Materials). Cortical regions involved in spatial item WM were identified by stronger univariate activations in the spatial item WM condition compared with the baseline condition in the item probe task (Awh et al., 1999; Curtis et al., 2005; Huang et al., 2016; Kang et al., 2011; Lee et al., 2008; Libby et al., 2014; McCarthy et al., 1996; Piefke et al., 2012; Toepper et al., 2010), the two-back task (Liao et al., 2012; Nagel et al., 2013; Yan et al., 2011), and the immediate serial recall task (Rotzer et al., 2009; Zhang et al., 2004). This mask was aligned to the Brodmann atlas, and 55 nodes were covered.
  Item probe tasks were used to investigate the neural correlates of the spatial item WM. With spatial locations as items, extensive regions in the frontal, temporal, and parietal lobes were found to be more active in the item probe conditions rather than the control condition, including the frontal eye field (FEF), supplementary eye field (SEF), MFG, pre-SMA, precuneus, superior and inferior parietal lobules, right STG, parahippocampal cortex, and posterior hippocampus (Awh et al., 1999; Curtis et al., 2005; Huang et al., 2016; Kang et al., 2011; Lee et al., 2008; Libby et al., 2014; McCarthy et al., 1996; Piefke et al., 2012; Toepper et al., 2010). The load effect in immediate serial recall tasks was also used as the index of the neural correlates of the spatial item WM. It was observed in the left DLPFC, right MFG, bilateral premotor cortex, right temporoparietal junction (TPJ), bilateral superior parietal lobule, and bilateral middle occipital gyrus (Rotzer et al., 2009; Zhang et al., 2004).
  The two-back paradigm was also used to investigate spatial item WM albeit the confounding involvement of the serial order monitoring. Given that spatial item WM and serial order WM are included in the same function-specific subnetwork, we included activated regions in this paradigm without further differentiation. The bilateral DLPFC, bilateral SMA, superior and inferior parietal lobules, precuneus, right ITG, and bilateral anterior insula were found activated (Liao et al., 2012; Nagel et al., 2013; Yan et al., 2011).

- **Spatial serial order WM**
  We reviewed task-based fMRI studies for the neural correlates of spatial serial order WM and created functional spherical ROIs based on reported peak activation coordinates (Table S5, Supplementary Materials). Cortical regions involved in spatial serial order WM were identified by stronger univariate activations in the serial order condition compared with the control or item condition in the order probe task (Davis et al., 2009; Rowe & Passingham, 2001) and the immediate serial recall task (Zhang et al., 2004). This mask was aligned to the Brodmann atlas, and 46 nodes were covered.
  In the order probe tasks, activities contrasted with the item condition were used to identify the neural correlates of the spatial serial order WM. Extensive regions in the frontal and parietal lobes were found to be more active in the order probe conditions than the item condition, including the FEF and SMA in the SFG, IFG, orbitofrontal cortex, paracingulate cortex, TPJ, SMG, and IPS (Davis et al., 2009; Rowe & Passingham, 2001). The order effect in the immediate serial recall of spatial locations was also used as the neural correlates for spatial serial order WM, which included the right PFC, right premotor, left superior parietal lobule, and right TPJ (Zhang et al., 2004).

- **Spatial attention**
  We reviewed task-based fMRI studies for the neural correlates of covert spatial attention orienting and created functional spherical ROIs based on reported peak activation coordinates (Table S6, Supplementary Materials).^8^ Cortical regions involved in spatial item WM were identified by stronger univariate activations in the orienting condition contrasted against the baseline condition in the Posner cueing task using peripheral exogenous cues (Kastner et al., 1999; Vandenberghe et al., 2001) and central endogenous cues (Corbetta et al., 1998, 2000, 2002; Hopfinger et al., 2000; Ikkai & Curtis, 2008; Munneke et al., 2010; Vandenberghe et al., 2001; Yantis et al., 2002). This mask was aligned to the Brodmann atlas, and 56 nodes were covered.
  In the Posner cueing paradigm, peripheral flash (Kastner et al., 1999; Vandenberghe et al., 2001) or symbols (e.g., arrows, words, shape, color; Corbetta et al., 1998, 2000, 2002; Hopfinger et al., 2000; Ikkai & Curtis, 2008; Munneke et al., 2010; Vandenberghe et al., 2001; Yantis et al., 2002) were used as cues to orient attention. Frontal and parietal regions were activated, including the FEF and SEF in the SFG, anteromedial SFG, MFG, precentral gyrus, bilateral IFG, IPS, superior and inferior parietal lobules, SMG, anterior insula-frontal operculum, precuneus, and posterior cingulate cortex. Temporal regions were activated too, including STG, STS, and ITG. Moreover, extensive regions in the visual cortex, such as the lingual gyrus in the middle occipital gyrus (MOG) and lateral occipital complex (LOC) in the occipital lobe, were also activated.

##### Modularity calculation

The modularity for each network was calculated with the Brain Connectivity Toolbox in MATLAB. The adjacency matrix for the network was thresholded to preserve the strongest 11.47% edges and then binarized.^9^ Thresholding can eliminate spurious connections and emphasize topological properties, and binarization could increase the signal-to-noise ratio and, in turn, improve community detection and modularity characterization (Chen & Deem, 2015; Yue et al., 2017). Afterward, the modularity of each network was defined as $M=\frac{1}{2e}\sum_{all modules}\sum_{i,j}({A}_{ij}-\frac{{a}_{i}{a}_{j}}{2e})$, where *i*, *j* are nodes within a module, *A*_*ij*_ is 1 if there is an edge between node *i* and node *j* and otherwise 0, the value of *a*_*i*_ = ∑_*j*_* A*_*ij*_ is the degree of node *i*, and *e* = ½ ∑_*i*_* a*_*i*_ is the total number of edges. Under certain partition of a network, modules are subsets of nodes within which the observed density of the network is greater than chance. A high-quality partition corresponds to internally dense, externally sparse, clearly delineated modules. The measure of modularity indexes the quality of the partition. We used Newman’s algorithm (Newman, 2006) to optimize the partition and maximize modularity.

#### Behavior and its relationship to modularity

In Experiment 2, we focused on the magnitude of the SPoARC effect, and thus only item probe tasks with consonants and colors in the behavioral battery were used. Unlike in Experiment 1, the SPoARC effect for Experiment 2 was calculated as a single-level measure. Reaction times of item probe tasks with both stimulus types were used as the dataset. After removing participant-level outliers and trial-level outliers in correct trials with positive probes (YES trials) (see item probe tasks in Experiment 1), the SPoARC effect magnitude was calculated for each participant as follows. We split the dataset by the responding hand, and RTs were linearly regressed on the serial position of the probe in each split dataset. The magnitude of the SPoARC effect was the linear slope of the right-hand subtracted by the left-hand dataset. A negative value suggests the presence of the SPoARC effect, and a smaller value (i.e., a larger absolute value) implies a larger SPoARC effect. The Pearson correlations between the magnitude of the SPoARC effect and the modularity value of different networks were calculated. Dependent correlation tests (Steiger, 1980) were used to assess whether this relationship in function-specific subnetworks was different from the whole brain network and its constituent networks.

A positive correlation suggests that less modular organization and greater interactivity within the network is associated with a larger SPoARC effect. To confirm that this relationship is specific and not merely a result of increased interactivity at any network scale, we also analyzed two other scales: the whole brain network and constituent networks. A significant positive correlation between the SPoARC effect and the modularity of the whole brain network would suggest that the brain’s overall interactivity, rather than confining to specific functions, supports spatialization. The critical evidence comes from the correlation of the SPoARC effect with two functions-specific subnetworks, which indicate whether interactions between hypothesized cognitive functions are the basis of spatialization. Conversely, a significant positive correlation with a constituent network does not directly explain its interaction with brain regions involved in other cognitive functions. Instead, it would only suggest that a less modular structure within the constituent network might allow for greater interaction with regions responsible for other functions in a larger-scale network, potentially contributing to the SPoARC effect.

### Results

#### Modularity of networks

The descriptive statistics for the modularity measures at different scales of networks are reported in Table 4. Details about each network and its modular organization are reported below.

**Table 4:** Descriptive statistics of modularity measures in different scales of networks

	mean	SD	min	max	skew	kurtosis
whole brain	0.46	0.05	0.34	0.55	− 0.28	− 0.43
vsowm + spatial attention	0.43	0.06	0.32	0.52	0.10	− 1.43
vsowm + siwm + ssowm	0.44	0.07	0.34	0.56	0.25	− 1.25
vsowm	0.43	0.07	0.31	0.55	0.16	− 0.94
spatial attention	0.46	0.07	0.35	0.57	− 0.03	− 1.34
siwm	0.48	0.06	0.38	0.58	− 0.06	− 1.50
ssowm	0.40	0.07	0.22	0.53	− 0.52	0.12

*N* = 25 for all variables. *vsowm* = verbal serial order working memory, *siwm* = spatial item working memory, *ssowm* = spatial serial order working memory

For the whole brain network, 84 BAs were used as nodes. When the graph density was 11.47% (top 400 edges), modularity values ranged from 0.34 to 0.55, with a mean of 0.46 and a standard deviation of 0.05. Individual differences were observed in the community organization. Most participants had four (10/25) or six modules (7/25), and fewer participants had five (3/25), seven (3/25), or three (2/25) modules.

For the verbal serial order WM + spatial attention function-specific subnetwork, 66 BAs were used as nodes. When the graph density was 11.47% (top 247 edges), modularity values ranged from 0.32 to 0.52, with a mean of 0.43 and a standard deviation of 0.06. Individual differences were observed in the community organization. Most participants had five (15/25) or six (6/25) modules, and fewer participants having four (3/25) or three (1/25) modules.

For the verbal serial order WM + spatial item WM + spatial serial order WM function-specific subnetwork, 72 BAs were used as nodes. When the graph density was 11.47% (top 294 edges), modularity values ranged from 0.34 to 0.56, with a mean of 0.44 and a standard deviation of 0.07. Individual differences were observed in the community organization. Most participants had six (11/25) or five (6/25) modules, and fewer participants had seven (4/25), four (2/25), or three or eight (both 1/25) modules.

The modularity values were also calculated for four constituent function-specific networks. For the verbal serial order WM network, 52 BAs were used as nodes. When the graph density was 11.47% (top 153 edges), modularity values ranged from 0.31 to 0.55, with a mean of 0.43 and a standard deviation of 0.07. Individual differences were observed in the community organization. Most participants had four or five (both 7/25), seven (6/25), or six (5/25) modules.

For the spatial attention network, 56 BAs were used as nodes. When the graph density was 11.47% (top 177 edges), modularity values ranged from 0.35 to 0.57, with a mean of 0.46 and a standard deviation of 0.07. Individual differences were observed in the community organization. Most participants had six (9/25), four (6/25), or five (6/25) modules, and fewer participants had seven (3/25) or eight (1/25) modules.

For the spatial item WM network, 55 BAs were used as nodes. When the graph density was 11.47% (top 171 edges), modularity values ranged from 0.38 to 0.58, with a mean of 0.48 and a standard deviation of 0.06. Individual differences were observed in the community organization. Most participants had five (10/25), four (8/25), or six (5/25) modules, and fewer participants had three or seven (both 1/25) modules.

For the spatial serial order WM network, 46 BAs were used as nodes. When the graph density was 11.47% (top 119 edges), modularity values ranged from 0.22 to 0.53, with a mean of 0.40 and a standard deviation of 0.07. Individual differences were observed in the community organization. Most participants had four (8/25), five, or six (both 6/25) modules, and fewer participants had seven (3/25) or three (2/25) modules.

#### Behavioral measures and correlation analyses

The mean SPoARC effect was − 23.18, with a standard deviation of 45.58. The range of the SPoARC effect was − 124.64 to 69.78. The skewness (− 0.25) and kurtosis (− 0.17) were less than 2, indicating that it was approximately univariate normally distributed (Kline, 1998; Ryu, 2011).

**Fig. 8:**
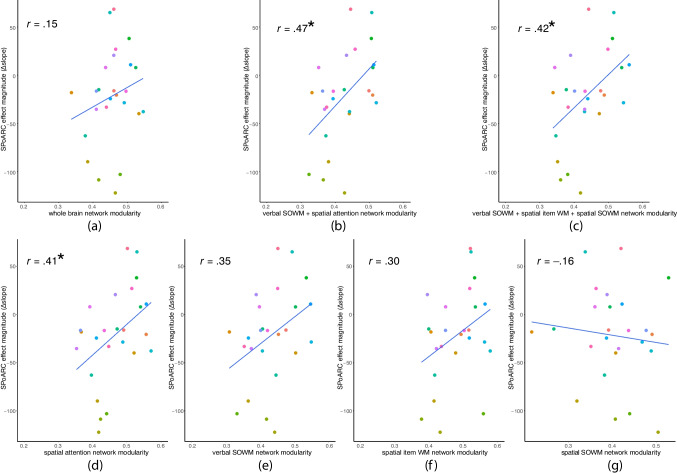
Relationship between the SPoARC effect and the modularity of** (a) **the whole brain network, **(b)** function-specific subnetwork: the verbal serial order WM + spatial attention network,** (c) **function-specific subnetwork: the verbal serial order WM + spatial item WM + spatial serial order WM network, and constituent networks of** (d) **spatial attention,** (e) **verbal serial order WM,** (f) **spatial item WM, and** (g) **spatial serial order WM. WM = working memory; SOWM = serial order working memory. The x-axis is the modularity of the corresponding network, with a lower value indicating a more interactive, less modular network. The y-axis is the magnitude of the SPoARC effect, with a smaller value indicating a larger SPoARC effect. *Significant correlation between the modularity and the magnitude of the SPoARC effect, in the direction that lower modularity is associated with larger SPoARC effect. Data points represent individual participants. Lines represent the linear fit of the SPoARC effect magnitude and the modularity

For the whole brain network, its modularity did not significantly correlate with the SPoARC effect, *r* = 0.15, *p* = 0.47 (Fig. 8a).

For the first function-specific subnetwork, the modularity of the verbal serial order WM + spatial attention network significantly correlated with the SPoARC effect magnitude (Fig. 8b, r = 0.47, *p* = 0.02). This positive association was expected, whereby the more crosstalk within verbal serial order WM and spatial attention network (reflected by lower modularity), the stronger the SPoARC effect is (indicated by a more negative value of between-hand slope difference). Furthermore, for its constituent networks (Figs. 8d and e), the larger magnitude of the SPoARC effect was significantly associated with a less modular organization of the spatial attention network (*r* = 0.41, *p* = 0.04), but not with such organization of the verbal serial order WM network (*r* = 0.35, *p* = 0.09). The verbal serial order WM + spatial attention network correlated with the magnitude of the SPoARC effect to a significantly larger extent than the verbal serial order WM network (*z* = 3.26, *p* < 0.001) and marginally larger extent than the spatial attention network (*z* = 1.51, *p* = 0.06), respectively. The advantage of a set of brain regions corresponding to multiple cognitive functions over its constituent networks was observed.

For the second function-specific subnetwork, a more crosstalk, less modular organization of the verbal serial order WM + spatial item WM + spatial serial order WM network was significantly associated with a stronger SPoARC effect (Fig. 8c, r = 0.42, *p* = 0.04), confirming experiment 1 from a neural perspective. For its constituent networks (Figs. 8e, f, and g), the magnitude of the SPoARC effect was not significantly correlated with modularity values of the verbal serial order WM network (*r* = 0.35, *p* = 0.09), the spatial item WM network (*r* = 0.30, *p* = 0.15), nor the spatial serial order WM network (*r* = − 0.16, *p* = 0.44). The verbal serial order WM + spatial item WM + spatial serial order WM network was correlated with the magnitude of the SPoARC effect to a significantly larger extent than the spatial item WM (*z* = 2.18, *p* = 0.01) and spatial serial order WM network (*z* = 7.01, *p* < 0.001) and to a marginally larger extent than the verbal serial order WM network (*z* = 1.53, *p* = 0.06). The advantage of a set of brain regions corresponding to interconnected cognitive functions over its constituent networks was observed.

One key contribution of the current study was to examine whether the network property modularity of a more refined function-specific subnetworks, compared with the whole brain network, is a better index for the individual differences in the SPoARC effect. Hence, we used the dependent correlation test (Steiger, 1980) to compare the correlations of modularity and behavior at different scales of networks. As expected, the correlation between the SPoARC effect and the modularity of the whole brain network was significantly smaller than the correlation with the modularity of the verbal serial order WM + spatial attention network (*z* = 4.96, *p* < 0.001) and the verbal serial order WM + spatial item WM + spatial serial order WM network (*z* = 4.47, *p* < 0.001). Furthermore, the correlation of the SPoARC effect and modularity values was comparable between the verbal serial order WM + spatial attention and the verbal serial order WM + spatial item WM + spatial serial order WM network (*z* = 1.74, *p* = 0.08), suggesting that the two function-specific subnetworks performed similarly in predicting how modularity related to individual differences in the SPoARC effect.

## Experiment 2 discussion

In this experiment, we investigated the mesoscale neural correlates of the SPoARC effect, specifically evaluating the graph-theoretic measure modularity of different networks. Twenty-five individuals participated in this study, with resting-state functional MRI data collected and single-level SPoARC effect magnitude estimated. We analyzed the network property at two scales, including the whole brain network and two function-specific subnetworks. Two function-specific subnetworks were the verbal serial order WM + spatial attention network and the verbal serial order WM + spatial item WM + spatial serial order WM network (motivated by behavioral results in Experiment 1). Modularity was used as the network property, which quantifies the balance between the level of modular structure and the interactions between modules within a network. Using resting-state functional connectivity, we investigated the modularity and its relationship to the individual differences of the SPoARC effect. We found that (1) the modularity of the whole brain network did not index the SPoARC effect; (2) the modularity values of function-specific subnetworks (the verbal serial order WM + spatial attention network and the verbal serial order WM + spatial item WM + spatial serial order WM network) indexed the SPoARC effect such that lower modularity values (i.e., a network having less within-module and more cross-module connections) were significantly associated with larger SPoARC effects. Moreover, the association with function-specific subnetworks was generally better than their corresponding constituent networks, implying that the function-specific subnetworks were the appropriate scale as the mesoscale neural correlates; (3) Importantly, the modularity of function-specific subnetworks predicted the magnitude of the SPoARC effect to a greater extent than the whole brain network, implying that the network-level neural correlates were not just simply interactions between brain regions but rather interactions between specific brain regions corresponding to certain cognitive functions. In addition, between two function-specific subnetworks, the verbal serial order WM + spatial attention network was comparably indicative of the SPoARC effect as the one consisting of verbal serial order WM and spatial WM. Overall, this investigation revealed that the modularity values of function-specific subnetworks, but not the whole brain network, were the mesoscale network-level neural correlates of the SPoARC effect, implying the potential involvement of spatial attention and spatial WM in the emergence of the SPoARC effect. Experiment 2 innovates the validity of the network-level index for behavior and elucidates how neural activity can infer cognitive theory.

Resting-state fMRI examines the intrinsic connectivity of brain regions through the synchronization of spontaneous neural activities in a “task-free” context. In contrast, task-based fMRI identifies brain regions involved in specific cognitive processes. In task-based fMRI studies, the neural correlate of the SPoARC effect refers to regions selectively activated for this particular function (Cristoforetti et al., 2022; Zhou et al., 2021). In the current study, the network-based, mesoscale neural correlates differ in nature from the task-based investigations. Here, the SPoARC effect was characterized by the degree of interactivity among brain regions involved in hypothesized functions, rather than by specific neural activation sites. This resting-state, network-based neural correlate provides a trait-like characterization of how these regions interact, potentially reflecting the integrated processing across various cognitive functions that contribute to the SPoARC effect.

There is an ongoing debate about whether functional maps derived from spontaneous activities are comparable to those from task-evoked activations, especially for complex behaviors and across individuals. Findings on their similarities are mixed (Park et al., 2020; Zhang et al., 2016). Another type of functional connectivity approach uses task-based residual time-course data to derive a network, showing similarities with both resting-state connectivity and task-related components (Tao & Rapp, 2019). Moving forward, it will be important to determine the extent to which resting-state and task-based approaches provide similar or divergent perspectives on this cognitive phenomenon, particularly considering the collective contribution of multiple functions. Converging evidence from all of these methods will offer useful and complementary insights into the relationship between behavior and the brain.

One limitation of Experiment 2 is the method used to identify neural regions involved in each constituent cognitive function. Although the literature review was carefully conducted, the regions included in function-specific subnetworks may still be constrained by the scope of the literature considered. Another potential risk associated with region identification is that regions involved in multiple functions might be included in one function-specific subnetwork but not the other. Consistent with previous studies (Awh & Jonides, 2001; Labar et al., 1999), we found considerable overlap between the two function-specific subnetworks. Because our analysis focuses on interactions within a set of nodes, the inclusion or exclusion of a single node is unlikely (although still possible) to drastically alter the correlation of one network with the SPoARC effect significant while leaving the other network nonsignificant. Our inclusive literature selection approach facilitates us to mitigate the potential omission of regions. Still, additional evidence, especially from other imaging methodologies, could further strengthen our findings. For example, considering individual differences in task-based activation locations, combining individualized function-specific regions defined through task-based localization with resting-state network-level connectivity could greatly enhance the robustness of the mesoscale network-level neural signature, if the findings converge with ours. Future studies that characterize how distinct and overlapping regions are connected within and between each constituent network would provide deeper insights into the interactions between these cognitive functions in the brain.

## General discussion

The SPoARC effect has been repeatedly observed in verbal WM performance. The mental whiteboard hypothesis (Abrahamse et al., 2014, 2017) proposes that the spatialization of WM gives rise to the SPoARC effect, suggesting that some spatial process underlies the SPoARC effect. However, what spatial process modulates the spatialization of verbal WM, and what drives this modulation, vary between individuals are largely unclear.

We reasoned that spatial WM could underpin the emergence of the SPoARC effect in that the ability to maintain spatial sequences in memory could be the ability to keep track of the temporary layout of WM information on the mental whiteboard. In addition, spatial attention, similar to the search process on the mental whiteboard, could be the mechanism that gives rise to the SPoARC effect. The effective scope of attention in space could be the capacity limit of how WM is spatialized on the mental whiteboard. Therefore, these spatial processes are viable candidates to ground the SPoARC effect. We conducted two experiments to investigate the spatial underpinning of the SPoARC effect, leveraging individual differences both in behavior and brain network organization.

The current study was designed to investigate the spatial modulation of verbal WM, via the mechanistic understanding of the SPoARC effect. In Experiment 1, we used a multilevel mixed-effects model to investigate the behavioral underpinning of the SPoARC effect. The SPoARC effect was gauged as the interaction of the responding hand with the serial position of the WM item in reaction times of item probe tasks. Participants performed a behavioral battery to examine three spatial attention capacities and four WM capacities. We systematically assessed how they relate to individual differences in the SPoARC effect in verbal WM. First, we provide evidence for the function of the SPoARC effect. Larger SPoARC effect was associated with higher capacity to maintain serial order information, but not item information, in verbal WM, suggesting that spatialization is one of the maintenance mechanisms for serial order WM. In addition, larger SPoARC effect was associated with higher capacity to maintain item information in spatial WM, revealing that the behavioral underpinning of the SPoARC effect is spatial item WM. Last, opposite directions have been found between the SPoARC effect and spatial serial order WM, where a smaller SPoARC effect was associated with higher serial order WM capacity, implying competition for cognitive resources or potential interference in their representational space.

In Experiment 2, we used the network neuroscience approach to assess the mesoscale neural correlates of the SPoARC effect. The SPoARC effect emerges through the interaction between verbal serial order WM and spatial processing, and the magnitude of the SPoARC effect is likely a reflection of the extent of such interaction. The mesoscale graph-theoretic metric modularity offers a well-suited tool for the neural correlates of the SPoARC effect in that it characterizes the interactivity between neural regions. Constructing networks based on resting-state functional connectivity, we examined the modularity of networks at two scales, including the whole brain network and function-specific subnetworks. Two function-specific subnetworks were defined as the joint network of verbal serial order WM and spatial attention or spatial WM, respectively. We found that the SPoARC effect is larger when the brain regions corresponding to verbal serial order WM have more interactions with brain regions involved with spatial attention as well as with spatial WM (for both item and serial order information). Critically, this association was not observed at the whole brain level and was weaker with their corresponding constituent networks. The results reveal that both spatial WM and spatial attention are involved in the neural mechanism of the SPoARC effect.

Together, two experiments offer a neurocognitive model of the SPoARC effect, unveiling the mechanism by which spatial processing modulates verbal WM. These empirical patterns offer concrete evidence for the behavioral and neural underpinnings of the mental whiteboard hypothesis.

Behavioral and neural evidence converged in terms of several mechanisms of the SPoARC effect.^10^ First, as expected, the SPoARC effect potentially serves as a mnemonic device for the maintenance of serial order information in verbal memoranda: when participants use spatialization for verbal memoranda more actively, reflected in larger SPoARC effects, they have higher verbal serial order WM capacities; when their neural regions engaged in verbal serial order WM have more interactions with neural regions engaged in spatial processing, either spatial attention or spatial WM, they exhibit larger SPoARC effects. These converging patterns suggest that spatialization serves a facilitatory role for verbal WM capacity for serial order information. Most WM theories assume certain rehearsal mechanisms, including subvocal/articulatory rehearsal, refreshing, and elaboration. These mechanisms are dedicated to an entire sequence (e.g., reciting the whole list silently, refreshing items sequentially), but they do not differentially specify serial order. Moreover, little evidence has been reported for actual improvement in WM performance induced by these rehearsal mechanisms (for review, see Oberauer, 2019). Spatialization involves a left-to-right search process on the “mental whiteboard,” similar to the refreshing mechanism, where attention is directed to individual items to maintain their heightened states. Another rehearsal mechanism, elaboration, enriches the to-be-remembered representation by adding meaning, such as creating visual images or forming a sentence with individual items. Spatialization is similar to elaboration in that it adds contextual details to the WM representation by linking spatial locations to the memorized information. However, spatialization is the first verbal WM rehearsal mechanism that supports only serial order information, despite resembling both refreshing and elaboration. The current study provides converging evidence from individual differences in both behavior and spontaneous neural activities that spatialization facilitates the performance of verbal serial order WM. Future studies are needed to confirm the causal role of spatialization in the improvement of verbal serial order WM, albeit the difficulty of inhibiting the automatic emergence of the SPoARC effect. Moreover, it is worth investigating how spatialization operates in addition to other rehearsal mechanisms in verbal WM.

Second, somewhat unexpectedly, our results reveal that spatial item WM underpins the SPoARC effect. Convergingly patterns were observed both behaviorally and neurally: when participants have higher spatial item WM capacities, they tend to exhibit larger SPoARC effects; when their neural regions engaged in spatial item WM have more interactions with those regions responsible for verbal and spatial serial order WM, they exhibit larger SPoARC effects. This suggests that our ability to maintain locations (i.e., spatial item information) at the external space in WM could overlap to some extent or be identical to the ability to maintain the internal space that the SPoARC effect occurs. Although domain-general processing is a long-standing debate in WM theories (Barrouillet & Camos, 2010; Morey & Mall, 2012), the dissociation between verbal and spatial WM is well-established (Basso et al., 1982; Hanley et al., 1991; Vallar & Baddeley, 1984). The SPoARC effect, specifically its facilitation of verbal serial order WM and its underpinning of spatial item WM, draws a connection between these two dissociated cognitive functions. Future work is needed to understand how spatial WM supports the spatialization of verbal WM and how to incorporate this connection in current WM models.

Third, spatial serial order WM interacts with the SPoARC effect both behaviorally and neurally: when participants have lower spatial serial order WM capacities, they tend to exhibit larger SPoARC effects; when their neural regions engaged in spatial serial order WM have more interactions with those regions responsible for verbal serial order WM and spatial item WM, they exhibit larger SPoARC effects. The neuroimaging results suggest that spatial serial order WM is active during the spatialization of verbal WM. Interestingly, the behavioral results indicate that its capacity is in the opposite direction as the magnitude of the SPoARC effect, which may be driven by the competition for limited cognitive resources or interference between the representations of spatial serial order WM and the mental whiteboard. A negative correlation implies that the spatial serial order WM mechanism does not underpin the SPoARC effect. Instead, we speculate that they have a “trade-off” relationship due to competition for shared cognitive resources. While these resources might not be used simultaneously (e.g., spatial serial order WM capacity being used in spatial WM tasks versus the SPoARC effect being measured in verbal WM tasks), the more automated spatialization is used for verbal WM tasks (as indicated by a larger SPoARC effect), the fewer resources can be allocated to spatial serial order WM, resulting in a lower span. It is also possible that the negative association indicates that the representations of the SPoARC effect and serial order in spatial WM are similar and therefore interfere with each other. The SPoARC effect arises when verbal WM information is laid out on the mental whiteboard, potentially allowing representations to entail all positional codes and strength-based gradients from both edges of the sequence. The serial order representation in WM is gradients based on both the start and the end for verbal sequences (Fischer-Baum & McCloskey, 2015) but is only a start-based gradient for spatial sequences (Fischer-Baum, 2018). For verbal sequences, similar representations for serial order and spatialization may enhance each other or converge in their computation. However, for spatial sequences, the serial order representation might be more malleable owing to its single anchor at the start of the sequence, making it more susceptible to disruption by the automatized spatialization process. That is, a more active spatialization process (reflected in a larger SPoARC effect) could induce stronger interference with the serial order representation for spatial sequences, leading to a lower serial order span in spatial WM tasks.

How and when does this interference happen? It is related to another unanswered question: does the SPoARC effect occur when holding a spatial location sequence in WM? The SPoARC effect has been observed only with verbal materials; for visual materials, it is present when materials have clear verbal labels or when participants use verbal labels as mnemonic strategies. However, it is not observed in spatial WM tasks (Ginsburg et al., 2017). During spatial WM tasks, space is used for both spatial WM sequences and the mental whiteboard. The spatialization is automatized, and when an effortful serial ordering process for spatial sequences is carried out, the spatialization is inhibited to some extent. Depending on the extent, the SPoARC effect could occur to a minor degree and is not able to be observed in RT or not occur at all. Future studies can use multivariate pattern analysis to decode whether the pattern of the SPoARC effect is present in spatial WM tasks in any part of the brain. Other measures for spatialization (e.g., eye movement tracking or embedded line bisection tasks; Antoine et al., 2017; Rinaldi et al., 2015) or explicit instruction to verbalize locations during WM tasks (Ginsburg et al., 2017) can also be used to enhance the SPoARC effect and assess whether it occurs in spatial WM tasks in a manner that is sensitive to manipulations.

While many of the results were consistent across the behavioral and neural experiments, discrepancies between the approaches were also observed. Specifically, the behavioral results of Experiment 1 suggest that spatial attention does not support the SPoARC effect. However, evidence from neural activity in Experiment 2 showed that the interaction between neural regions of spatial attention and verbal serial order WM predicted the magnitude of the SPoARC effect. These seemingly conflicting results highlight the importance of using different methodologies to gather multimodal converging evidence. The spatial attention process is likely involved in the emergence of the SPoARC effect (as indicated by its association with network properties of regions related to spatial attention), but it does not determine the extent to which a WM sequence is spatialized on the mental whiteboard (as evidenced by the lack of a moderation effect of spatial attention in the behavioral data).

We recommend caution in interpreting the role of spatial attention in spatialization. First, the lack of association in Experiment 1 might be due to the specific paradigms used, as variations in paradigm design can strongly influence the presence and magnitude of the cueing effect of exogenous and endogenous orienting in space (Chica et al., 2014). It is worth exploring how the relationship between attention orienting and the SPoARC effect varies across different levels of exogenous and endogenous orienting capacities. Additionally, other types of spatial attention, such as the ability to resize (Eriksen & James, 1986; Lawrence et al., 2020) or reshape the effective field of view (Jefferies & Di Lollo, 2015), the ability to divide attention to multiple discrete regions (Müller et al., 2003), or alternative breadth of attention measures (Goodhew & Plummer, 2019; Irons & Leber, 2016), could be considered as candidates for the spatial underpinning of the SPoARC effect. If systematic investigation reveals an association between spatial attention capacity and the SPoARC effect, the converging neuroimaging and behavioral results would suggest that spatial attention is not only involved in the manifestation of the SPoARC effect but may also underpin the spatialization of verbal WM.

Conversely, the association observed in our neuroimaging study might be coincidental. Experiment 2 was exploratory and aimed to confirm behavioral findings from Experiment 1 using a network neuroscience approach. In Experiment 2, we found that higher interactivity within the spatial attention and verbal serial order WM network correlated with a greater degree of spatialization (as indicated by a larger SPoARC effect), suggesting the involvement of spatial attention. However, because of the correlational and confirmatory nature of Experiment 2, the small sample size, and the potential limitations of defining the network scope based on the literature review, further investigation is necessary. Given the variability in neural signals among individuals, a larger sample using individualized activation-based localizations might be needed to confirm whether spatial attention is truly involved in spatialization in the brain. Additionally, further research is needed to determine the specific stage of WM at which spatial attention is engaged and how this involvement occurs. If future neuroimaging studies using different methodologies do not find an association between spatial attention and the SPoARC effect, it may indicate that, despite the similarities between orienting attention in space and retrieving information from the “mental whiteboard,” spatial attention might not be directly connected to this effect, either behaviorally or neurologically.

The current study innovates in three ways. First, we provide insights into the implication of network neuroscience in cognitive psychology. For instance, the involvement of spatial attention in the SPoARC effect is inferred from the scope of function-specific subnetworks. Using function-specific subnetworks to predict variability in the SPoARC effect allows us to test hypotheses about cognitive functions giving rise to the phenomenon, offering new ways to infer the underlying cognitive architecture. Additionally, the current study provides a new perspective on how to study the neural correlates of tasks that require the interaction of cognitive processes. In Experiment 2, we found that modularity values of two function-specific subnetworks, but not the whole brain network, were predictive of the magnitude of the SPoARC effect. These results further confirm that the network-level neural correlate of the SPoARC effect is not a product of the positive manifold or extensive coverage of the brain. The variability in the SPoARC effect did not correlate nonselectively with individual differences in modularity values of all or any subset of brain regions. Rather, this graph-theoretic metric is only indicative when brain regions corresponding to related cognitive functions are included in the network. This finding further validates the potential of using network-level properties as the neural correlate of a cognitive function, converging with prior studies (Bassett et al., 2011, 2013; Braun et al., 2015; Gallen et al., 2016; Kitzbichler et al., 2011; Ramos-Nuñez et al., 2017; Stevens et al., 2012; Tao & Rapp, 2019; Westphal et al., 2017; Yue et al., 2017), especially the ones where specific subnetworks better index cognition rather than a global network (Gallen et al., 2016; Iordan et al., 2018). The primary approach for investigating neural correlates of a cognitive function is to identify specialized brain regions based on univariate activation or multivariate decoding. Using graph-theoretic analysis, multiple brain regions are considered as a network, allowing regions to be grouped by specialized functions as in the activation-based analysis but also incorporating the interactions between such specialized structures. Network-level neural correlates could be a useful candidate to characterize cognitive functions, especially those with an interactive nature (e.g., multisensory integration, language production, comprehension, reasoning). Network analysis at more granularities could also be used in future studies, such as specific operations of each module (connector versus hub, core versus periphery), or other level of network measures (e.g., small-worldness, node degree).

Second, we enrich the mental whiteboard hypothesis (Abrahamse et al., 2014, 2017), which implies that space is repurposed to encode verbal WM representation. Through the individual differences in the SPoARC effect and its relationship to variability in behavior and brain network structure, we elaborate the foundation of the mental whiteboard hypothesis. When laying out verbal WM information in space, serial order representations are enhanced, and improved serial order WM capacity is observed. This spatialization process is supported by spatial item WM capacity—the capacity by which spatial locations (whereabouts, not when they occur) are maintained in WM. The recoding in space also involves spatial attention, which is likely the process by which attention is oriented or allocated for rehearsal or retrieval of the WM information in the repurposed space. However, spatial attention is not a bottleneck for this process. The extent to which space is taxed for verbal WM does not depend on how efficiently attention is oriented on the mental whiteboard. Additionally, the spatialization is likely to interfere with serial order representations in spatial WM. Verbal serial order WM is closely associated with the capacity to maintain serial order information in spoken production (Acheson & MacDonald, 2009; Tian et al., 2025), reading (Bogaerts et al., 2016; Martinez Perez et al., 2012), and spelling (Binamé & Poncelet, 2016), as well as linguistic behavior, such as speech perception (Brady et al., 1983) and vocabulary acquisition (Leclercq & Majerus, 2010; Majerus et al., 2006a, b, 2008b; Majerus & Boukebza, 2013), and nonlinguistic behavior, such as ordinal processing in alphabets (Attout & Majerus, 2018; Attout et al., 2014a), musical memory (Gorin et al., 2016, 2018a, b; Gorin & Majerus, 2019), and arithmetic (Attout & Majerus, 2015, 2018; Attout et al., 2014a, b). Therefore, a richer understanding of the mental whiteboard hypothesis allows us to better characterize verbal serial order WM and where it situates in relationship to other cognitive processes.

Last, we provide insights into the architecture of WM. The fractionations in WM are corroborated by selective interactions with the SPoARC effect. The item versus serial order fractionation in verbal and spatial domains (Jarrold, 2017; Majerus, 2019) is supported by the results that the SPoARC effect selectively interacts with verbal serial order but not item WM and that spatial item and serial order WM capacities interact with the SPoARC effect in opposite directions. Additionally, the fractionation between serial order WM in verbal and spatial domains (Tian et al., 2022, 2025) is confirmed by the results that serial order WM capacities in verbal and spatial domains are associated with the magnitude of the SPoARC effect in opposite directions. Moreover, the selective facilitation of the spatialization to verbal—but not spatial—serial order WM could be a possible source of domain-specificity. While confirming the fractionations in WM, our findings pose new challenges to current WM models. Although dissociated, a connection between verbal and spatial WM is also needed to account for the spatialization of verbal WM.

## Supplementary information

Below is the link to the electronic supplementary material.

Supplementary file1 (DOCX 17.2 MB)

## Data Availability

The code and scripts of analyses can be found at https://osf.io/7sjum/.

## Footnotes

1 While there is substantial evidence that item and order processing are distinct in verbal and spatial WM, we do not suggest that WM capacities for item and order processing, or for verbal and spatial domains, are entirely separate. In fact, these capacities significantly overlap. Item and order processing in WM may rely on distinct processes and yet still share a common resource (Guitard et al., 2022; Majerus, 2008). Similarly, some findings suggest that a domain-general WM mechanism may support both domains with a specialized store for verbal memoranda, rather than strictly domain-specific mechanisms (Morey & Mall, 2012). In this study, we consider WM under the domain-specific and process-specific framework, while also acknowledging the overlap between these processes.

2 Multilevel models had comparable results when *altL* included and excluded the participant with exposure to sign language. We report results with *altL*, including the sign language to account for the exposure to all languages that could affect spatialization. For the model results with *altL* = 1 when participants acquired right-to-left languages (excluding sign languages), see Supplementary Materials Table S2.

3 Two other theory-driven four-way CLI (*vsowm**×ssowm**×Hand**×Position* and *viwm**×vsowm**×Hand**×Position*) also were assessed separately, reflecting the concept that serial order WM interacted differently with the SPoARC effect in verbal and spatial domains and that verbal item and serial order WM interacted differently with the SPoARC effect, respectively. None of them were significant, so we did not report these two parallel models.

4 The same multilevel model was fitted in the subset of behavioral dataset without 25 participants with neuroimaging data; same moderation effects on the SPoARC effect magnitude were found.

5 The functional network has been found to exhibit consistent trends using other parcellation schemes in previous modularity studies (Yue et al., 2017).

6 We did not concatenate three runs’ time series for each participant’s adjacency matrix. Because of the possible discontinuity in the BOLD signals at the point of concatenation, spurious correlations could be found between regions.

7 Note that a node in the function-specific mask could cover brain regions smaller than the scope of an entire BA, depending on the coverage of spheres from each involved cognitive function.

8 Although having distinct behavioral patterns, the exogenous and endogenous orienting processes share largely overlapping neural correlates in the frontoparietal regions (Chica et al., 2011; 2013; Meyer et al., 2018). Given that the discrepancies between the neural correlates were small and the neural correlates of exogenous orienting were less extensive than the endogenous orienting, I kept two processes together as spatial attention in the current study.

9 Previous research has shown that the modularity values of networks are consistent with different graph densities (Bonomo et al., 2022; Ramos-Nuñez et al., 2017; Yue et al., 2017).

10 Although these inferences are supported by converging patterns, they are based on correlational, not causal, relationships.

## Declarations

### Open practices statement

The data and materials for all experiments are available at OSF (https://osf.io/7sjum/). Neither of the experiments was preregistered.
