# Supplementary material for: The role of spatial processing in verbal serial order working memory
Source: Cogn Affect Behav Neurosci. 2025 Jan 15;25(1):210–39. doi: 10.3758/s13415-024-01240-6 (PMC11805787; doi:10.3758/s13415-024-01240-6)
Supplement: Supplementary file 1 — Supplementary file1 (DOCX 17.2 MB) [file 13415_2024_1240_MOESM1_ESM.docx]

**Supplementary materials**

Table S1

Nested Model Comparison

| Model | *npar* | AIC | BIC | deviance | χ^2^ | ∆*df* | *p* (>χ^2^) |
| --- | --- | --- | --- | --- | --- | --- | --- |
| null | 3 | 149128 | 149150 | 149122 |  |  |  |
| 1 | 8 | 148570 | 148628 | 148554 | 567.827 | 5 | < .001 |
| 2 | 20 | 148570 | 148715 | 148530 | 23.835 | 12 | .02 |
| 3 | 29 | 147962 | 148172 | 147904 | 626.112 | 9 | < .001 |
| 4 | 37 | 147962 | 148231 | 147888 | 15.7 | 8 | .046 |

*Note*. All statistics were from FIML estimation of each model. *npar* = the number of estimated parameters in the model. χ^2^ is the difference of deviance statistics between two nested models. ∆*df* is degree of freedom for the chi-squared distribution, which equals to the difference of degrees of freedom between two nested models. Null = model with fixed and random intercept; 1 = null model plus three L1 predictors and two L1 interactions; 2 = model 1 plus contextual effect of seven L2 cognitive and five L2 demographic predictors; 3 = model 2 plus variance components for L1 *Task*, *Hand× Position, and Task× Hand× Position;* 4 = model 3 plus eight cross-level interactions.

**Supplementary materials**

The Presence of Individual Differences in The SPoARC Effect

Four options convergingly supported the presence of by-subject random slope of *Hand× Position*. That is, the SPoARC effect (i.e., the relationship between RTs and *Hand× Position*) varied depending on participants.

The first line of support came from the likelihood ratio test (i.e., the chi-squared test of the deviance difference) of nested FIML estimated models. As shown in Table S1, Model 3 fit the data better than Model 2, χ^2^(9) = 626.11, *p* < .001, suggesting nonzero random slopes of *Hand× Position*, *Task*, and *Task× Hand× Position*. More specifically, when only adding the random slope of *Hand× Position* to Model 2, two parameters (i.e., the variance of by-subject *Hand× Position* slope τ_33_ and the intercept–*Hand× Position* covariance τ_03_) were added the model fit was improved significantly, χ^2^(2, *N* = 160) = 30.47, *p* < .001.

The second line of support came from the simulation of the log-likelihood ratio (i.e., the difference in deviance values) between Model 2 and Model 3 under the null (Crainiceanu & Ruppert, 2004). The proportion of simulated log-likelihood ratio values larger than the observed one (626.11) was 0, suggesting the presence of three by-subject variance components. The same result remained when the simulation was based on the change induced by Model 2 adding only the random slope of *Hand× Position* (with the observed value being 30.47).

The third line of support came from the nonparametric bootstrap simulation of the confidence interval for τ_33_ (Carpenter et al., 2003). With 1500 runs of simulation, the 95% bootstrap confidence interval for the variance of by-subject *Hand× Position* τ_33_ was [334.32, 1351.72]. The confidence interval excluding zero suggested the presence of the variance component.

Lastly, in the FIML estimation of Model 3, the 95% parametric confidence interval for the standard error of *u_3j_* based on the likelihood profile was [24.61, 38.69]. The confidence interval for $\sqrt{\tau_{33}}$ excluding zero suggested the presence of the variance component.

In sum, the parametric profile-based confidence interval for $\sqrt{\tau_{33}}$ and the nonparametric bootstrapping confidence interval for τ_33_ did not contain zero, and the log-likelihood ratio tests with both chi-squared test and the mixture-of-chi-squared test (Crainiceanu & Ruppert, 2004) were significant after adding the variance component for *Hand× Position*. Results based on our data convergingly suggest the presence of the by-subject variance component of the slope of *Hand× Position*.

**Supplementary materials**

In this line of supplementary analysis, *altL* = 1 represents that a participant has acquired a language with a right-to-left reading/writing direction, while *altL* = 0 applies to those who have learned only language(s) with a left-to-right reading/writing direction. Although sign language is expressed in space, it does not have a specific directionality; thus, *altL* = 0 for participants who can use sign language.

Table S2

Multilevel Mixed Effect Model for Reaction Time in Item Probe Tasks

|  | Est (SE) | df | *t* | *p* |
| --- | --- | --- | --- | --- |
| **Fixed effects** |  |  |  |  |
| **L1** |  |  |  |  |
| Intercept (𝛾_00_) | 1200.54(283.43) | 149.37 | 4.24 | <.001 |
| *Hand* (𝛾_10_) | 32.17(6.6) | 9845.02 | 4.88 | <.001 |
| *Position* (𝛾_20_) | -37.37(1.92) | 9830.69 | -19.49 | <.001 |
| *Hand× Position* (𝛾_30_) | -23.27(4.02) | 255.14 | -5.79 | <.001 |
| *Task* (𝛾_40_) | -69.62(13.94) | 158.61 | -5.00 | <.001 |
| *Task× Hand× Position* (𝛾_50_) | 11.27(2.17) | 151.47 | 5.19 | <.001 |
| **L2 cognitive variables** |  |  |  |  |
| *boa* (𝛾_01_) | 1.04(1.34) | 147.33 | 0.78 | .44 |
| *exo* (𝛾_02_) | 1.2(0.49) | 148.14 | 2.43 | .02 |
| *endo* (𝛾_03_) | -0.61(0.45) | 147.47 | -1.37 | .17 |
| *viwm* (𝛾_05_) | 44.96(147.84) | 147.34 | 0.30 | .76 |
| *vsowm* (𝛾_06_) | 191.13(113.96) | 147.33 | 1.68 | .10 |
| *siwm* (𝛾_04_) | 40.49(133.59) | 147.90 | 0.30 | .76 |
| *ssowm* (𝛾_07_) | -111.35(113.05) | 147.20 | -0.99 | .33 |
| **L2 control variables** |  |  |  |  |
| handedness (𝛾_08_) | 118.66(58.59) | 147.19 | 2.03 | .04 |
| altL (𝛾_09_) | 71.36(85.75) | 148.40 | 0.83 | .41 |
| school (𝛾_010_) | 1.17(138.98) | 149.06 | 0.01 | .99 |
| education years (𝛾_011_) | -28.43(15.17) | 146.98 | -1.87 | .06 |
| gender (𝛾_012_) | 67.64(33.13) | 147.06 | 2.04 | .04 |
| **Cross-level interactions** |  |  |  |  |
| *boa× Hand× Position* (𝛾_31_) | 0.13(0.1) | 145.19 | 1.41 | .16 |
| *exo× Hand× Position* (𝛾_32_) | -0.02(0.03) | 146.31 | -0.46 | .65 |
| *endo× Hand× Position* (𝛾_33_) | 0.02(0.03) | 147.34 | 0.59 | .56 |
| *viwm× Hand× Position* (𝛾_35_) | 9.72(10.36) | 144.46 | 0.94 | .35 |
| *vsowm× Hand× Position* (𝛾_36_) | -19.37(7.86) | 143.22 | -2.47 | .01 |
| *siwm× Hand× Position* (𝛾_34_) | -24.26(10.86) | 146.83 | -2.23 | .03 |
| *ssowm× Hand× Position* (𝛾_37_) | 17.85(7.95) | 143.30 | 2.25 | .03 |
| *siwm× ssowm× Hand× Position* (𝛾_347_) | -100.49(41.29) | 152.71 | -2.43 | .02 |
| **Random effects (variance components)** |  |  |  |  |
| L1 variance (𝜎^2^) | 76098.60 |  |  |  |
| Intercept (L2) variance (𝜏_00_) | 100100.10 |  |  |  |
| *Hand×Position* (L2) variance (𝜏_33_) | 1001.70 |  |  |  |
| *Task* (L2) variance (𝜏_44_) | 26262.60 |  |  |  |
| *Task×Hand×Position* (L2) variance (𝜏_55_) | 357.30 |  |  |  |
| Intercept–*Hand×Position* covariance (𝜏_03_) | -5407.42 |  |  |  |
| Intercept–*Task* (L2) covariance (𝜏_04_) | -41532.07 |  |  |  |
| Intercept–*Task×Hand×Position* cov (𝜏_05_) | 3408.47 |  |  |  |
| *Hand×Position*–*Task* (L2) covariance (𝜏_34_) | 2256.85 |  |  |  |
| *Hand×Position*–*Task×Hand×Position* cov (𝜏_35_) | -574.26 |  |  |  |
| *Task*–*Task×Hand×Position* covariance (𝜏_45_) | -1531.47 |  |  |  |
| **Model summary (FIML)** |  |  |  |  |
| ICC | 0.29 |  |  |  |
| Deviance statistic | 147888 |  |  |  |
| AIC | 147962 |  |  |  |
| BIC | 148230 |  |  |  |
| Number of estimated parameters | 37 |  |  |  |

*Note.* L1 = Level 1; L2 = Level 2. L1 sample size = 10435 and L2 sample size = 160. REML = restricted maximum likelihood estimation. Est: unstandardized estimations of the fixed effects 𝛾*_ij_* in the REML estimated model; SE = standard errors. *t*-statistics were computed as the ratio of each regression coefficient divided by its standard error. *: *p* < .05 in two-tailed *t*-test with the Satterthwaite approximation for their significances. FIML = full information maximum likelihood estimation; all fit indices (the deviance statistic, AIC, and BIC) were calculated from the FIML estimated model. *boa* = breadth of attention, *exo* = efficiency of exogenous orienting, *endo* = efficiency of endogenous orienting, *viwm* = verbal item working memory capacity, *vsowm* = verbal serial order working memory capacity, *siwm* = spatial item working memory capacity, *ssowm* = spatial serial order working memory capacity.

**Supplementary materials**

Table S3

*Peak Activations of Clusters Engaged in Verbal Serial Order WM*

| brain area | x | y | z | reference | condition |
| --- | --- | --- | --- | --- | --- |
| left MTG | -66 | -28 | 2 | Attout (2014) encoding Table 4 | letter: OP > null |
| left postcentral gyrus | -64 | -16 | 20 | Majerus (2009) Table 7 | nonword OP > face OP |
| left STG superior temporal gyrus | -62 | -30 | 6 | Majerus (2009) Table 7 | nonword OP > face OP |
| left ventral motor cortex (vMC) | -62 | -3 | 8 | Kalm & Norris (2014) encoding | nonword ISR decoding |
| left IFG | -62 | 14 | 20 | Majerus & Poncelet (2006) Table 2 | word: OP > IP |
| left SMG | -59 | -27 | 17 | Kalm & Norris (2014) encoding | nonword ISR decoding |
| left IFG | -58 | 5 | 9 | Kalm & Norris (2014) encoding | nonword ISR decoding |
| left IFG | -58 | 12 | 14 | Majerus & Poncelet (2006) Table 2 | word: OP > IP |
| left postcentral | -56 | -2 | 20 | Attout (2014) encoding Table 4 | letter: OP > null |
| left middle frontal gyrus | -55 | 10 | 40 | Majerus & Poncelet (2006) | ROI |
| left superior planum temporale SPT | -54 | -39 | 19 | Kalm & Norris (2014) encoding | nonword ISR decoding |
| left precentral | -54 | 2 | 46 | Majerus & Poncelet (2006) Table 2 | word: OP > IP |
| left IFG | -54 | 6 | 18 | Majerus (2009) Table 7 | nonword OP > face OP |
| left middle frontal gyrus (premotor) | -54 | 12 | 36 | Henson (2000) Table 3 | letter: OP > IP |
| left IPL | -52 | -21 | 40 | Kalm & Norris (2014) encoding and recall | nonword ISR decoding |
| left precentral gyrus | -52 | 10 | 14 | Majerus & Belayachi (2008) encoding Table 2 | word: OP > IP |
| left inf occipital gyrus | -50 | -64 | -14 | Attout (2014) encoding Table 4 | letter: OP > null |
| left precentral gyrus | -50 | 0 | 46 | Attout (2014) retrieval Table 1 | letter: OP > null |
| left IFG | -50 | 6 | 19 | Kalm & Norris (2014) encoding | nonword ISR decoding |
| left mFG/IFG | -48 | 0 | 39 | Oztekin (2009) | letter: OP > IP |
| left IPL | -46 | -50 | 52 | Majerus & Poncelet (2006) Table 2 | word: OP > IP |
| left IFG | -46 | 6 | 32 | Majerus & Belayachi (2008) encoding Table 2 | word: OP > IP |
| left mid-fusiform gyrus | -44 | -58 | -2 | Majerus (2009) Table 7 | nonword OP > face OP |
| left ant IPS | -42 | -42 | 40 | Attout (2014) | ROI |
| left ant IPS (postcentral by IPL) | -38 | -40 | 50 | Majerus & Belayachi (2008) encoding Table 2 | word: OP > IP |
| left ant IPS | -36 | -44 | 43 | Marshuetz & Smith (2000) Table4 | letter: OP > IP |
| left ant IPS | -36 | -42 | 32 | Attout (2014) encoding Table 4 | letter: OP > null |
| left IFG | -36 | 18 | 26 | Attout (2014) retrieval Table 1 | letter: OP > null |
| left IFG | -36 | 20 | -14 | Majerus & Belayachi (2008) retrieval Table 3 | word: OP > IP |
| left IPS | -34 | -50 | 48 | Majerus & Poncelet (2006) | ROI |
| left ant IPS | -34 | -46 | 38 | Attout (2014) retrieval Table 1 | letter: OP > null |
| left inf parietal cortex; left PPC | -34 | -46 | 46 | Marshuetz & Smith (2000) Table3 | letter: OP > IP |
| left pos IPS/left inf parietal cortex | -34 | -46 | 46 | Marshuetz & Smith (2000) Table4; Marshuetz (2006) Fig 3 | letter: OP > IP |
| left precentral | -34 | -4 | 52 | Majerus & Poncelet (2006) Table 2 | word: OP > IP |
| left insula | -34 | 22 | 0 | Majerus & Belayachi (2008) encoding Table 2 | word: OP > IP |
| left occipital gyrus | -30 | -96 | -6 | Oztekin (2009) | letter: OP > IP |
| left middle occipital gyrus | -30 | -93 | 12 | Henson (2000) Table 3 | letter: OP > IP |
| left SFG / pre-SMA | -30 | 8 | 58 | Attout (2019) Table 2 | word: OP > IP |
| left IFG | -30 | 27 | 3 | Oztekin (2009) | letter: OP > IP |
| left SFG | -28 | 6 | 70 | Attout (2014) retrieval Table 1 | letter: OP > null |
| left fusiform | -27 | -78 | -18 | Henson (2000) Table 3 | letter: OP > IP |
| left IPS | -27 | -63 | 39 | Oztekin (2009) | letter: OP > IP |
| left IPS | -27 | -48 | 45 | Oztekin (2009) | letter: OP > IP |
| left pos IPS | -26 | -62 | 46 | Attout (2014) | ROI |
| left pos IPS | -24 | -70 | 44 | Attout (2014) encoding Table 4 | letter: OP > null |
| left superior parietal gyrus | -24 | -70 | 45 | Attout (2014) retrieval Table 1 | letter: OP > null |
| left PPC; left pos IPS | -24 | -60 | 42 | Marshuetz & Smith (2000) Table2 | letter: OP > IP |
| left PPC; left pos IPS | -24 | -60 | 42 | Majerus & Poncelet (2006) | ROI |
| left superior parietal gyrus | -24 | -51 | 51 | Henson (2000) Table 3 | letter: OP > IP |
| left hippocampus | -24 | -18 | -12 | Attout (2014) encoding Table 4 | letter: OP > null |
| left SFG | -24 | 4 | 58 | Majerus & Belayachi (2008) encoding Table 2 | word: OP > IP |
| left lingual gyrus | -14 | -92 | -10 | Attout (2014) retrieval Table 1 | letter: OP > null |
| left inferior occipital cortex | -12 | -92 | -10 | Majerus & Belayachi (2008) retrieval Table 3 | word: OP > IP |
| left precuneus | -8 | -66 | 66 | Majerus & Poncelet (2006) Table 2 | word: OP > IP |
| left precuneus | -6 | -64 | 56 | Majerus & Poncelet (2006) Table 2 | word: OP > IP |
| left SFG | -4 | -48 | -22 | Attout (2014) encoding Table 4 | letter: OP > null |
| left SMA/ SFG | -3 | 6 | 60 | Oztekin (2009) | letter: OP > IP |
| left SMA | -2 | -2 | 74 | Attout (2014) encoding Table 4 | letter: OP > null |
| left medial SMA/ACC | -2 | 18 | 54 | Majerus & Belayachi (2008) encoding Table 2 | word: OP > IP |
| right precuneus | 6 | -64 | 56 | Majerus & Poncelet (2006) Table 2 | word: OP > IP |
| right precuneus | 8 | -66 | 66 | Majerus & Poncelet (2006) Table 2 | word: OP > IP |
| right medial frontal gyrus/ACC | 8 | 42 | 26 | Majerus & Belayachi (2008) retrieval Table 3 | word: OP > IP |
| right calcarine sulcus | 14 | -88 | 2 | Attout (2014) retrieval Table 1 | letter: OP > null |
| right lingual gyrus | 24 | -94 | -10 | Attout (2014) encoding Table 4 | letter: OP > null |
| right sup parietal cortex; right PPC | 26 | -56 | 52 | Marshuetz & Smith (2000) Table1 | letter: OP > IP |
| right sup parietal cortex | 26 | -56 | 52 | Marshuetz (2006) Fig 3 | letter: OP > IP |
| right middle frontal gyrus | 26 | 18 | 56 | Majerus & Poncelet (2006) Table 2 | word: OP > IP |
| right occipital gyrus | 27 | -99 | -9 | Oztekin (2009) | letter: OP > IP |
| right superior parietal gyrus | 27 | -54 | 51 | Henson (2000) Table 3 | letter: OP > IP |
| right pos IPS | 28 | -58 | 40 | Attout (2014) | ROI |
| right IPS | 28 | -50 | 38 | Majerus, Belayachi 2008 | ROI |
| right pos IPS | 28 | -42 | 48 | Marshuetz & Smith (2000) Table0 | letter: OP > IP |
| right supeior BA 6 | 28 | 2 | 56 | Marshuetz & Smith (2000) Table4; Marshuetz (2006) Fig 3 | letter: OP > IP |
| right SFG | 28 | 10 | 54 | Attout (2019) Table 2 | word: OP > IP |
| right fusiform | 30 | -75 | -15 | Henson (2000) Table 3 | letter: OP > IP |
| right PPC | 30 | -62 | 42 | Marshuetz & Smith (2000) Table1 | letter: OP > IP |
| right hippocampus | 30 | -44 | 4 | Majerus & Belayachi (2008) encoding Table 2 | word: OP > IP |
| right pos IPS | 32 | -70 | 50 | Attout (2014) encoding Table 4 | letter: OP > null |
| right middle IPS | 32 | -44 | 45 | Marshuetz & Smith (2000) Table2 | letter: OP > IP |
| right IFG | 32 | 20 | -16 | Majerus & Belayachi (2008) retrieval Table 3 | word: OP > IP |
| right middle frontal gyrus | 32 | 58 | 8 | Majerus & Poncelet (2006) Table 2 | word: OP > IP |
| right middle IPS | 36 | -44 | 39 | Marshuetz & Smith (2000) Table3 | letter: OP > IP |
| right insula | 36 | 22 | -2 | Majerus & Belayachi (2008) encoding Table 2 | word: OP > IP |
| right IPS (postcentral by IPL) | 38 | -48 | 44 | Majerus & Poncelet (2006) | ROI |
| right IPL | 38 | -33 | 45 | Kalm & Norris (2014) encoding and recall | nonword ISR decoding |
| right IPS | 40 | -42 | 44 | Majerus, Belayachi 2008 | ROI |
| right middle frontal gyrus | 40 | 42 | 30 | Majerus & Belayachi (2008) encoding Table 2 | word: OP > IP |
| right middle occipital gyrus | 42 | -87 | 0 | Henson (2000) Table 3 | letter: OP > IP |
| right IPS | 42 | -46 | 50 | Attout (2019) Table 2 | word: OP > IP |
| right middle frontal gyrus | 42 | 28 | 40 | Majerus & Belayachi (2008) retrieval Table 3 | word: OP > IP |
| right middle frontal gyrus | 42 | 36 | 30 | Attout (2014) encoding Table 4 | letter: OP > null |
| right IPS | 44 | -56 | 44 | Majerus, Belayachi 2008 | ROI |
| right IPS | 44 | -40 | 42 | Majerus & Poncelet (2006) Table 2 | word: OP > IP |
| right ant IPS | 44 | -40 | 44 | Attout (2014) | ROI |
| right IPS | 44 | -39 | 50 | Majerus & Poncelet (2006) | ROI |
| right ant IPS | 44 | -35 | 48 | Marshuetz & Smith (2000) Table4 | letter: OP > IP |
| right ant IPS | 46 | -52 | 48 | Majerus & Belayachi (2008) retrieval Table 3 | word: OP > IP |
| right DLPFC | 46 | 39 | 7 | Marshuetz & Smith (2000) Table4; Marshuetz (2006) Fig 3 | letter: OP > IP |
| right IPS | 48 | -40 | 44 | Majerus & Poncelet (2006) Table 2 | word: OP > IP |
| right IFG | 52 | 16 | 2 | Majerus & Poncelet (2006) Table 2 | word: OP > IP |
| right SPT | 57 | -28 | 12 | Kalm & Norris (2014) recall | nonword ISR decoding |
| right SMG | 59 | -17 | 15 | Kalm & Norris (2014) encoding | nonword ISR decoding |
| right IFG, oper | 59 | 18 | 20 | Kalm & Norris (2014) encoding | nonword ISR decoding |
| right vMC | 61 | 6 | 25 | Kalm & Norris (2014) recall | nonword ISR decoding |

Table S4

*Peak Activations of Clusters Engaged in Spatial Item WM*

| brain area | x | y | z | reference | contrast |
| --- | --- | --- | --- | --- | --- |
| left precentral gyrus | -54 | 3 | 42 | Piefke (2012) Table4 | loc IP |
| left VLPFC/IFG, oper | -54 | 9 | 9 | Toepper (2010) encoding contrast 1 Table 1, Table3 | loc IP |
| left DLPFC/mFG | -51 | 6 | 36 | Toepper (2010) encoding contrast 1 Table 1, Table3 | loc IP |
| left IFJ | -48 | 2 | 31 | Huang (2016) | loc IP |
| left IFG, trian | -48 | 33 | 27 | Piefke (2012) Table3 | loc IP |
| left IFG, oper | -45 | 9 | 27 | Piefke (2012) Table3 | loc IP |
| left IFG, trian | -45 | 24 | 30 | Piefke (2012) Table3 | loc IP |
| left IPL | -44 | -27 | 58 | Liao (2012) Table 4 | loc 1-back (ctrl+schizophrenia group) |
| left pos DLPFC | -44 | 8 | 27 | Kang (2011) Table 2 | loc pattern complete (ctrl + schizophrenia group) |
| left ant DLPFC | -44 | 32 | 16 | Kang (2011) Table 2 | loc pattern complete (ctrl + schizophrenia group) |
| left fusiform gyrus | -42 | -72 | -13 | Kang (2011) Table 2 | loc pattern complete (ctrl + schizophrenia group) |
| left dorsal pos mFG | -40 | 1 | 43 | Yan (2011) Table 1 | loc 2-back (item+order) |
| left IFG,orbs | -40 | 19 | -12 | Kang (2011) Table 2 | loc pattern complete (ctrl + schizophrenia group) |
| left postcentral gyrus | -39 | -45 | 60 | Toepper (2010) encoding contrast 1 Table 1, Table3 | loc IP |
| left precentral gyrus | -39 | 0 | 57 | Piefke (2012) Table3 | loc IP |
| left middle occipital gyrus | -36 | -90 | 9 | Rotzer (2009) Table 2 | loc ISR (control group) |
| left LOG/ITG | -36 | -82 | 13 | Huang (2016) | loc IP |
| left LOG | -36 | -80 | 24 | Libby,Hunnula,& Ranganath (2014) | scene IP |
| left IPL | -36 | -37 | 43 | Huang (2016) | loc IP |
| left precentral gyrus | -36 | -3 | 60 | Piefke (2012) Table4 | loc IP |
| left DLPFC | -36 | 61 | 4 | Nagel (2013) Table 4 | loc 2-back > dots 2-back |
| left mFG | -35 | 11 | 58 | Liao (2012) Table 4 | loc 1-back (ctrl+schizophrenia group) |
| left inferior parietal lobule | -34 | 60 | 45 | Yan (2011) Table 1 | loc 2-back (item+order) |
| left VLPFC / insula | -33 | 18 | 0 | Toepper (2010) encoding contrast 1 Table 1, Table3 | loc IP |
| left ant insula | -33 | 19 | 1 | Nagel (2013) Table 4 | loc 2-back > dots 2-back |
| left ant IFG | -33 | 36 | 6 | Rotzer (2009) Table 2 | loc ISR (control group) |
| left inferior parietal lobule | -30 | -45 | 42 | Piefke (2012) Table4 | loc IP |
| left VLPFC / insula | -30 | 24 | 6 | Toepper (2010) encoding contrast 1 Table 1, Table3 | loc IP |
| left IPS | -27 | -48 | 45 | Rotzer (2009) Table 2 | loc ISR (control group) |
| left precentral gyrus / premotor | -27 | -3 | 57 | Toepper (2010) encoding contrast 1 Table 1, Table3 | loc IP |
| left IPS | -24 | -73 | 31 | Huang (2016) | loc IP |
| left SFG | -24 | -3 | 54 | Piefke (2012) Table3 | loc IP |
| left FEF | -24 | -1 | 58 | Huang (2016) | loc IP |
| left SFG | -21 | -3 | 54 | Piefke (2012) Table4 | loc IP |
| left SPL | -18 | -64 | 52 | Huang (2016) | loc IP |
| left superior parietal lobule | -15 | -69 | 54 | Piefke (2012) Table3 | loc IP |
| left superior parietal lobule | -15 | -69 | 54 | Piefke (2012) Table4 | loc IP |
| left V1 | -14 | -98 | 4 | Kang (2011) Table 2 | loc pattern complete (ctrl + schizophrenia group) |
| left PPC | -13 | -69 | 51 | Kang (2011) Table 2 | loc pattern complete (ctrl + schizophrenia group) |
| left precuneus | -12 | -66 | 63 | Piefke (2012) Table4 | loc IP |
| left precuneus | -9 | -72 | 54 | Piefke (2012) Table3 | loc IP |
| left lingual gyrus | -8 | -90 | 6 | Libby,Hunnula,& Ranganath (2014) | scene IP |
| left SEF | -5 | -4 | 64 | Curtis (2005) | loc IP |
| left SFG | -3 | -8 | 50 | Yan (2011) Table 1 | loc 2-back (item+order) |
| left pre-SMA | -3 | 12 | 51 | Piefke (2012) Table3 | loc IP |
| left medial pre-SMA, mSFG | -3 | 20 | 43 | Huang (2016) | loc IP |
| left posterior cingulate | -2 | 55 | 24 | Yan (2011) Table 1 | loc 2-back (item+order) |
| left medial frontal gyrus | -1 | 21 | 60 | Liao (2012) Table 4 | loc 1-back (ctrl+schizophrenia group) |
| right ventral medial frontal gyrus | 2 | 51 | 17 | Yan (2011) Table 1 | loc 2-back (item+order) |
| right dACC | 4 | 12 | 42 | Curtis (2005) | loc IP |
| right medial frontal gyrus | 4 | 21 | 60 | Liao (2012) Table 4 | loc 1-back (ctrl+schizophrenia group) |
| right precuneus | 6 | -66 | 54 | Piefke (2012) Table3 | loc IP |
| right superior parietal lobule | 15 | -69 | 60 | Piefke (2012) Table3 | loc IP |
| right SPL/precuneus | 15 | -69 | 60 | Piefke (2012) Table4 | loc IP |
| right SPL/precuneus | 15 | -63 | 66 | Piefke (2012) Table4 | loc IP |
| right pos cingulate cortex | 15 | -43 | 7 | Lee (2008) | loc IP (control>schizo) |
| right superior parietal lobule | 16 | -62 | 61 | Nagel (2013) Table 4 | loc 2-back > dots 2-back |
| right cuneus | 18 | -60 | 21 | Piefke (2012) Table3 | loc IP |
| right V1 | 21 | -96 | 6 | Kang (2011) Table 2 | loc pattern complete (ctrl + schizophrenia group) |
| right SPL | 21 | -69 | 54 | Piefke (2012) Table4 | loc IP |
| right PPC | 21 | -69 | 55 | Kang (2011) Table 2 | loc pattern complete (ctrl + schizophrenia group) |
| right superior parietal lobule | 21 | -63 | 66 | Toepper (2010) encoding contrast 1 Table 1, Table3 | loc IP |
| right SPL | 21 | -61 | 58 | Huang (2016) | loc IP |
| right mFG | 24 | -6 | 66 | Rotzer (2009) Table 2 | loc ISR (control group) |
| right SFG | 24 | -3 | 57 | Toepper (2010) encoding contrast 1 Table 1, Table3 | loc IP |
| right dorsal mFG | 26 | 5.9 | 56 | Yan (2011) Table 1 | loc 2-back (item+order) |
| right middle occipital gyrus | 27 | -60 | 57 | Rotzer (2009) Table 2 | loc ISR (control group) |
| right FEF | 27 | -7 | 52 | Huang (2016) | loc IP |
| right SFG | 27 | 0 | 54 | Piefke (2012) Table4 | loc IP |
| right mFG | 28 | 51 | 8 | Lee (2008) | loc IP (control>schizo) |
| right precuneus | 28 | 60 | 45 | Yan (2011) Table 1 | loc 2-back (item+order) |
| right IPS | 30 | -82 | 31 | Huang (2016) | loc IP |
| right FEF | 30 | -5 | 64 | Curtis (2005) | loc IP |
| right mFG | 32 | 8 | 61 | Nagel (2013) Table 4 | loc 2-back > dots 2-back |
| right VLPFC / insula | 33 | 18 | 0 | Toepper (2010) encoding contrast 1 Table 1, Table3 | loc IP |
| right VLPFC / insula | 33 | 24 | 6 | Toepper (2010) encoding contrast 1 Table 1, Table3 | loc IP |
| right ant insula | 35 | 22 | -4 | Nagel (2013) Table 4 | loc 2-back > dots 2-back |
| right mFG | 35 | 43 | 14 | Liao (2012) Table 4 | loc 1-back (ctrl+schizophrenia group) |
| right middle occipital gyrus | 36 | -78 | 33 | Piefke (2012) Table4 | loc IP |
| right SMG | 36 | -39 | 42 | Piefke (2012) Table4 | loc IP |
| right IFG, orbs | 37 | 22 | -13 | Kang (2011) Table 2 | loc pattern complete (ctrl + schizophrenia group) |
| right fusiform gyrus | 38 | -82 | -11 | Kang (2011) Table 2 | loc pattern complete (ctrl + schizophrenia group) |
| right inferior parietal lobule | 39 | -48 | 54 | Piefke (2012) Table4 | loc IP |
| right SPL | 42 | -39 | 65 | Liao (2012) Table 4 | loc 1-back (ctrl+schizophrenia group) |
| right ant mFG | 42 | 35 | 7 | Huang (2016) | loc IP |
| right precentral gyrus | 44 | -2 | 36 | Yan (2011) Table 1 | loc 2-back (item+order) |
| right SFG | 45 | 43 | 9 | Lee (2008) | loc IP (control>schizo) |
| right middle occipital gyrus | 48 | -78 | -15 | Rotzer (2009) Table 2 | loc ISR (control group) |
| right LOG/ITG | 48 | -64 | -8 | Huang (2016) | loc IP |
| right IPL | 48 | -31 | 43 | Huang (2016) | loc IP |
| right IFJ | 48 | 5 | 28 | Huang (2016) | loc IP |
| right pos DLPFC | 48 | 8 | 26 | Kang (2011) Table 2 | loc pattern complete (ctrl + schizophrenia group) |
| right ant DLPFC | 48 | 37 | 12 | Kang (2011) Table 2 | loc pattern complete (ctrl + schizophrenia group) |
| right ITG | *54* | *-60* | *-12* | Piefke (2012) Table3 | loc IP |
| right VLPFC/IFG, oper | 54 | 9 | 21 | Toepper (2010) encoding contrast 1 Table 1, Table3 | loc IP |
| right ITG | 57 | -59 | -15 | Kang (2011) Table 2 | loc pattern complete (ctrl + schizophrenia group) |
| right STG | 58 | -3 | -11 | Lee (2008) | loc IP (control>schizo) |
| right inferior temporal gyrus | 60 | -52 | -14 | Nagel (2013) Table 4 | loc 2-back > dots 2-back |
| right DLPFC/mFG | 60 | 9 | 36 | Toepper (2010) encoding contrast 1 Table 1, Table3 | loc IP |

Table S5

*Peak Activations of Clusters Engaged in Spatial Serial Order WM*

| brain area | x | y | z | reference | contrast |
| --- | --- | --- | --- | --- | --- |
| left TPJ | -66 | -40 | 10 | Davis (2009) expt2 | loc OP |
| left TPJ | -66 | -38 | 24 | Davis (2009) expt2 | loc OP |
| left SMG | -60 | -42 | 34 | Davis (2009) expt 1 | loc OP |
| left TPJ | -58 | -54 | 6 | Davis (2009) expt 1 | loc OP |
| left SMG | -54 | -36 | 34 | Davis (2009) expt2 | loc OP |
| left TPJ | -50 | -48 | 10 | Davis (2009) expt2 | loc OP |
| left SMG | -50 | -44 | 38 | Davis (2009) expt 1 | loc OP |
| left TPJ | -50 | -42 | 20 | Davis (2009) expt2 | loc OP |
| left TPJ | -48 | -46 | 24 | Davis (2009) expt 1 | loc OP |
| left mFG | -48 | 32 | 18 | Rowe (2001) | loc OP |
| left TPJ | -46 | -44 | 20 | Davis (2009) expt 1 | loc OP |
| left IPS | -44 | -46 | 52 | Davis (2009) expt 1 | loc OP |
| left FEF | -44 | 6 | 44 | Davis (2009) expt2 | loc OP |
| left IPS | -40 | -42 | 54 | Davis (2009) expt2 | loc OP |
| left inferior frontal gyrus | -40 | 8 | 24 | Rowe (2001) | loc OP |
| left FEF | -36 | 6 | 56 | Davis (2009) expt2 | loc OP |
| left TPJ | -34 | -18 | 12 | Davis (2009) expt2 | loc OP |
| left orbitofrontal PFC | -32 | 24 | -13 | Rowe (2001) | loc OP |
| left BA 9 mFG | -24 | 10 | 50 | Rowe (2001) | loc OP |
| left prestriate cortex V2 | -10 | -100 | 0 | Rowe (2001) | loc OP |
| left medial parietal (precuneus) | -10 | 70 | 54 | Rowe (2001) | loc OP |
| left paracingulate cortex | -2 | 24 | 42 | Rowe (2001) | loc OP |
| right paracingulate cortex | 2 | 24 | 42 | Rowe (2001) | loc OP |
| right precuneus | 4 | -66 | 44 | Rowe (2001) | loc OP |
| right prestriate cortex V2 | 16 | -94 | 2 | Rowe (2001) | loc OP |
| right FEF | 16 | 4 | 70 | Davis (2009) expt2 | loc OP |
| right FEF | 20 | 16 | 46 | Davis (2009) expt2 | loc OP |
| right frontal pole | 20 | 54 | 12 | Davis (2009) expt 1 | loc OP |
| right SMA | 22 | 4 | 62 | Davis (2009) expt 1 | loc OP |
| right SMA | 26 | 2 | 62 | Davis (2009) expt 1 | loc OP |
| right SMA | 26 | 8 | 42 | Davis (2009) expt 1 | loc OP |
| right SMA | 26 | 10 | 46 | Davis (2009) expt 1 | loc OP |
| right FEF | 26 | 14 | 54 | Davis (2009) expt 1 | loc OP |
| right IFL | 28 | 48 | 2 | Davis (2009) expt 1 | loc OP |
| right BA 9 mFG | 30 | 10 | 54 | Rowe (2001) | loc OP |
| right orbitofrontal PFC | 34 | 24 | -12 | Rowe (2001) | loc OP |
| right IPS | 36 | -8 | 54 | Davis (2009) expt2 | loc OP |
| right middle frontal gyrus | 36 | 58 | 12 | Davis (2009) expt 1 | loc OP |
| right IFG | 36 | 58 | 12 | Davis (2009) expt 1 | loc OP |
| right SMA (in MFG) | 42 | 6 | 40 | Davis (2009) expt 1 | loc OP |
| right mFG | 42 | 38 | 30 | Rowe (2001) | loc OP |
| right IFL | 44 | 0 | -12 | Davis (2009) expt2 | loc OP |
| right IFG | 44 | 20 | 6 | Davis (2009) expt 1 | loc OP |
| right IFL | 44 | 32 | -6 | Davis (2009) expt 1 | loc OP |
| right inferior parietal cortex | 48 | -36 | 42 | Rowe (2001) | loc OP |
| right inferior frontal gyrus | 48 | 14 | 12 | Rowe (2001) | loc OP |
| right IFL | 50 | 40 | -10 | Davis (2009) expt 1 | loc OP |
| right pos inf temporal cortex | 52 | -62 | 8 | Davis (2009) expt 1 | loc OP |
| right IFL | 52 | 20 | 12 | Davis (2009) expt2 | loc OP |
| right IFL | 52 | 38 | -10 | Davis (2009) expt2 | loc OP |
| right IFG | 54 | 36 | -4 | Davis (2009) expt2 | loc OP |
| right TPJ | 58 | -40 | 24 | Davis (2009) expt 1 | loc OP |
| right TPJ | 60 | -52 | 16 | Davis (2009) expt 1 | loc OP |
| right IPS | 62 | -40 | 36 | Davis (2009) expt 1 | loc OP |
| right IFG | 63 | 33 | 23 | Davis (2009) expt2 | loc OP |
| right TPJ | 64 | -50 | 14 | Davis (2009) expt 1 | loc OP |
| right TPJ | 66 | -44 | 20 | Davis (2009) expt 1 | loc OP |

Table S6

*Peak Activations of Clusters Engaged in Spatial Attention*

| brain area | x | y | z | reference | function | cue |
| --- | --- | --- | --- | --- | --- | --- |
| left STG | -56 | -24 | 8 | Hopfinger (2000) | endo | arrow |
| left IFG, operculum | -53 | 14 | -5 | Vandenberghe, Gitelman, Parrish, & Mesulam (2011) | endo | color |
| left lateral SFG | -52 | 32 | 16 | Hopfinger (2000) | endo | arrow |
| left ant IPS (AG) | -44 | -64 | 32 | Hopfinger (2000) | endo | arrow |
| left pos IPS (SMG) | -44 | -48 | 32 | Hopfinger (2000) | endo | arrow |
| left FEF | -42 | 6 | 51 | Kastner (1999) Table 1 | exo | dot |
| left IFG,orb | -39 | 43 | -6 | Corbetta,Kincade, & Shulman (2002) | endo | arrow |
| left IPL | -38 | -28 | 64 | Kastner (1999) Table 1 | exo | dot |
| left dorsalateral sup precentral sulcus | -37 | -12 | 51 | Ikkai & Curtis (2008) | endo | shape |
| left LOG (lateral occipiral gyrus) | -36 | -76 | 4 | Hopfinger (2000) | endo | arrow |
| left ant IPS (IPL) | -36 | -47 | 46 | Ikkai & Curtis (2008) | endo | shape |
| left dorsal precentral*(mFG) | -36 | -5 | 48 | Corbetta (1998) | endo | arrow |
| left mFG (*postcentral) | -36 | 32 | 27 | Corbetta (1998) | endo | arrow |
| left mFG | -32 | 16 | 48 | Hopfinger (2000) | endo | arrow |
| left TEO | -31 | -70 | -21 | Kastner (1999) Table 1 | exo | dot |
| left mFG | -31 | 38 | 27 | Kastner (1999) Table 1 | exo | dot |
| left V4 | -30 | -85 | -25 | Kastner (1999) Table 1 | exo | dot |
| left insula | -28 | -8 | 20 | Hopfinger (2000) | endo | arrow |
| left IFG | -28 | 31 | -4 | Yantis (2002) | endo | arrow |
| left IPS | -27 | -78 | 45 | Kastner (1999) Table 1 | exo | dot |
| left sup precentral sulcus/ SFS(mFG*precentral) | -27 | -9 | 48 | Corbetta (1998) | endo | arrow |
| left IPS/TOS (transverse occipital sulcus) | -25 | -76 | 27 | Corbetta (1998) | endo | arrow |
| left ant sup IPS | -25 | -53 | 56 | Corbetta (1998) | endo | arrow |
| left dorsalmedial sup precentral sulcus | -24 | -12 | 58 | Ikkai & Curtis (2008) | endo | shape |
| left pos IPS | -18 | -60 | 68 | Corbetta (1998) | endo | arrow |
| left SPL | -17 | -61 | 61 | Kastner (1999) Table 1 | exo | dot |
| left SFG | -16 | 48 | 36 | Hopfinger (2000) | endo | arrow |
| left V2/VP | -13 | -87 | -18 | Kastner (1999) Table 1 | exo | dot |
| left SPL | -12 | -56 | 56 | Hopfinger (2000) | endo | arrow |
| left SEF (mFG) | -6 | -19 | 54 | Ikkai & Curtis (2008) | endo | shape |
| left precuneus | -5 | -71 | 34 | Corbetta,Kincade, & Shulman (2002) | endo | arrow |
| left cingualte sulcus | -5 | 18 | 45 | Vandenberghe, Gitelman, Parrish, & Mesulam (2011) | endo | color |
| left V1 | -4 | -89 | 12 | Kastner (1999) Table 1 | exo | dot |
| left precuneus | -4 | -71 | 40 | Corbetta & Kincade (2000) | endo | arrow |
| left pos medial frontal gyrus(*SFG) | -4 | 5 | 60 | Corbetta (1998) | endo | arrow |
| left pos cingulate | 0 | -40 | 44 | Hopfinger (2000) | endo | arrow |
| right SEF | 1 | 5 | 64 | Kastner (1999) Table 1 | exo | dot |
| right anteromedial SFG | 1 | 60 | 14 | Vandenberghe & Gitelman (2011) | exo | dot |
| right SEF | 3 | -11 | 55 | Ikkai & Curtis (2008) | endo | shape |
| right pos cingulate | 4 | -44 | 32 | Hopfinger (2000) | endo | arrow |
| right precuneus | 7 | -75 | 34 | Corbetta,Kincade, & Shulman (2002) | endo | arrow |
| right SPL | 8 | -40 | 56 | Hopfinger (2000) | endo | arrow |
| right precuneus | 9 | -75 | 40 | Corbetta & Kincade (2000) | endo | arrow |
| right pos medial frontal gyrus(*SFG) | 13 | -5 | 72 | Corbetta (1998) | endo | arrow |
| right SPL | 14 | -57 | 62 | Yantis (2002) | endo | arrow |
| right dorsalmedial sup precentral sulcus | 18 | -11 | 61 | Ikkai & Curtis (2008) | endo | shape |
| right SPL | 19 | -58 | 64 | Kastner (1999) Table 1 | exo | dot |
| right pos sup IPS | 20 | -63 | 61 | Corbetta (1998) | endo | arrow |
| right mFG | 20 | 8 | 48 | Hopfinger (2000) | endo | arrow |
| right insula | 24 | -8 | 12 | Hopfinger (2000) | endo | arrow |
| right ant IPS (SPL) | 27 | -61 | 52 | Ikkai & Curtis (2008) | endo | shape |
| right LOG (middle) | 28 | -80 | 0 | Hopfinger (2000) | endo | arrow |
| right ant IPS | 28 | -56 | 63 | Corbetta (1998) | endo | arrow |
| right MOG | 30 | -90 | 14 | Corbetta (1998) | endo | arrow |
| right IPS/TOS | 30 | -76 | 33 | Corbetta (1998) | endo | arrow |
| right lateral lingual gyrus | 32 | -85 | -11 | Corbetta (1998) | endo | arrow |
| right middle precentral gyrus | 32 | -2 | 25 | Yantis (2002) | endo | arrow |
| right sup precentral sulcus/ SFS | 33 | 3 | 62 | Corbetta (1998) | endo | arrow |
| right ant insula-FO | 33 | 23 | -2 | Corbetta,Kincade, & Shulman (2002) | endo | arrow |
| right mFG | 35 | 31 | 24 | Corbetta,Kincade, & Shulman (2002) | endo | arrow |
| right mFG | 35 | 39 | 36 | Kastner (1999) Table 1 | exo | dot |
| right pos IPS (SMG) | 36 | -44 | 32 | Hopfinger (2000) | endo | arrow |
| right superior precentral gyrus | 37 | -3 | 52 | Corbetta,Kincade, & Shulman (2002) | endo | arrow |
| right IFG,orb | 37 | 39 | -4 | Corbetta,Kincade, & Shulman (2002) | endo | arrow |
| right ant IPS | 39 | -47 | 48 | Corbetta,Kincade, & Shulman (2002) | endo | arrow |
| right FEF | 39 | -3 | 57 | Kastner (1999) Table 1 | exo | dot |
| right inf precentral sulcus | 39 | 2 | 33 | Ikkai & Curtis (2008) | endo | shape |
| right ventral precentral sulculs*IFG | 39 | 3 | 29 | Corbetta (1998) | endo | arrow |
| right IPL | 42 | -30 | 62 | Kastner (1999) Table 1 | exo | dot |
| right pos IFS | 42 | 15 | 38 | Ikkai & Curtis (2008) | endo | shape |
| right ant IPS | 43 | -44 | 53 | Corbetta & Kincade (2000) | endo | arrow |
| right ant IPS (AG) | 44 | -60 | 36 | Hopfinger (2000) | endo | arrow |
| right IOG | 45 | -78 | 6 | Corbetta (1998) | endo | arrow |
| right STS | 45 | -43 | 8 | Corbetta (1998) | endo | arrow |
| right dorsal precentral sulcus | 45 | -2 | 47 | Corbetta (1998) | endo | arrow |
| right MT/MST | 47 | -69 | 10 | Corbetta (1998) | endo | arrow |
| right dorsalateral sup precentral sulcus | 47 | -2 | 46 | Ikkai & Curtis (2008) | endo | shape |
| right inf temporal gyrus (pos) | 48 | -59 | -3 | Yantis (2002) | endo | arrow |
| right STG (ant) | 48 | -12 | 8 | Hopfinger (2000) | endo | arrow |
| right mFG (*postcentral) | 49 | 35 | 30 | Corbetta (1998) | endo | arrow |
| right IPL | 51 | -29 | 29 | Yantis (2002) | endo | arrow |
| right SMG | 53 | -49 | 30 | Corbetta,Kincade, & Shulman (2002) | endo | arrow |
| right STG | 55 | -44 | 28 | Ikkai & Curtis (2008) | endo | shape |
| right STG | 57 | -45 | 12 | Corbetta,Kincade, & Shulman (2002) | endo | arrow |
| right IPL | 58 | -48 | 33 | Corbetta & Kincade (2000) | endo | arrow |
| right STG | 62 | -45 | 12 | Corbetta & Kincade (2000) | endo | arrow |
| right FO (*precentral) | 62 | 7 | 19 | Corbetta (1998) | endo | arrow |
| right IPL, SMG | 66 | -50 | 26 | Vandenberghe & Gitelman (2011) | exo | dot |

*Figure S1*. Function-specific networks. (a) the verbal serial order WM + spatial attention network and (b) the verbal serial order WM + spatial item WM + spatial serial order WM network. Each color indicates the category to which the region belongs.
